# Deep-learning microscopy image reconstruction with quality control reveals second-scale rearrangements in RNA polymerase II clusters

**DOI:** 10.1093/pnasnexus/pgac065

**Published:** 2022-05-23

**Authors:** Hamideh Hajiabadi, Irina Mamontova, Roshan Prizak, Agnieszka Pancholi, Anne Koziolek, Lennart Hilbert

**Affiliations:** HIDSS4Health - Helmholtz Information and Data Science School for Health, 76131, Karlsruhe/Heidelberg, Germany; Institute of Biological and Chemical Systems, Department of Biological Information Processing, Karlsruhe Institute of Technology, 76344, Eggenstein-Leopoldshafen, Germany; KASTEL – Institute of Information Security and Dependability, Karlsruhe Institute of Technology, 76131, Karlsruhe, Germany; Institute of Biological and Chemical Systems, Department of Biological Information Processing, Karlsruhe Institute of Technology, 76344, Eggenstein-Leopoldshafen, Germany; Institute of Biological and Chemical Systems, Department of Biological Information Processing, Karlsruhe Institute of Technology, 76344, Eggenstein-Leopoldshafen, Germany; Institute of Biological and Chemical Systems, Department of Biological Information Processing, Karlsruhe Institute of Technology, 76344, Eggenstein-Leopoldshafen, Germany; KASTEL – Institute of Information Security and Dependability, Karlsruhe Institute of Technology, 76131, Karlsruhe, Germany; Institute of Biological and Chemical Systems, Department of Biological Information Processing, Karlsruhe Institute of Technology, 76344, Eggenstein-Leopoldshafen, Germany; Zoological Institute, Department of Systems Biology and Bioinformatics, Karlsruhe Institute of Technology, 76131, Karlsruhe, Germany

**Keywords:** image processing, reliable deep learning, fluorescence microscopy, gene regulation, transcription

## Abstract

Fluorescence microscopy, a central tool of biological research, is subject to inherent trade-offs in experiment design. For instance, image acquisition speed can only be increased in exchange for a lowered signal quality, or for an increased rate of photo-damage to the specimen. Computational denoising can recover some loss of signal, extending the trade-off margin for high-speed imaging. Recently proposed denoising on the basis of neural networks shows exceptional performance but raises concerns of errors typical of neural networks. Here, we present a work-flow that supports an empirically optimized reduction of exposure times, as well as per-image quality control to exclude images with reconstruction errors. We implement this work-flow on the basis of the denoising tool Noise2Void and assess the molecular state and 3D shape of RNA polymerase II (Pol II) clusters in live zebrafish embryos. Image acquisition speed could be tripled, achieving 2-s time resolution and 350-nm lateral image resolution. The obtained data reveal stereotyped events of approximately 10 s duration: initially, the molecular mark for recruited Pol II increases, then the mark for active Pol II increases, and finally Pol II clusters take on a stretched and unfolded shape. An independent analysis based on fixed sample images reproduces this sequence of events, and suggests that they are related to the transient association of genes with Pol II clusters. Our work-flow consists of procedures that can be implemented on commercial fluorescence microscopes without any hardware or software modification, and should, therefore, be transferable to many other applications.

Significance Statement Light microscopy is subject to unavoidable performance trade-offs. For observation of live biological samples, image acquisition speed can only be increased in exchange for a lowered signal quality, or an increased rate of photo-damage. These limitations can be partially compensated for by denoising after acquisition. Denoising based on deep learning performs especially well, but denoising errors have caused concern. We present a pragmatic work-flow that enables quality control of denoised images. We illustrate the applicability of this work-flow by assessing RNA polymerase II clusters in live zebrafish embryos, revealing coordinated changes in the molecular state and cluster shape. Our observations point toward the activation of genes over the course of 10 s during which they visit a polymerase cluster.

## Introduction

Light microscopy is one of the most central tools of biological research, whether a biologist aims to get the first glimpse of a given cellular process or to quantitatively test the validity of hypotheses (1). A specific area of application is the visualization of fluorescently labeled molecules. The design of such experiments is subject to inherent limitations (2, 3), requiring a trade-off between acquisition speed, signal-to-noise ratio (SNR), and prevention of photo-damage to the specimen (4). These parameters cannot be optimized separately. For instance, to increase acquisition speed, exposure time must be reduced, leading to lower SNR (5,6). SNR can be recovered by, for example, increased power of the light used to excite fluorescence in the sample, resulting however in increased photo-damage.

While the experimental parameters during acquisition are subject to firm trade-off relationships, computational processing of images after acquisition can recover image quality. These approaches allow, for example, a further reduction of exposure times followed by computational reconstruction of low-SNR images. Conventional approaches for reconstruction of low-SNR images include projection methods (7), deconvolution filters (8, 9), and denoising methods (10,11). In the past decade, deep-learning methods have become widely used in a variety of image processing applications, often outperforming conventional approaches (12). In biological microscopy, deep learning has been successfully used for image classification (13–15), segmentation (16,17), and restoration (18–21). Initial deep learning approaches used standard deep networks to restore fluorescence microscopy images, requiring training data sets of matched low-quality and high-quality images. For example, networks can be trained on a reference data set with high SNR (“ground truth”), so as to restore matched images with low SNR (“noisy data”) (22). One obstacle to the wide-spread application of such reconstruction approaches is the requirement for matched high-quality training data (23,24). These data are laborious or sometimes even impossible to obtain in a fashion that is sufficiently matched to noisy data. An alternative is provided by Noise2Noise (n2n) techniques, which enable the training of deep networks from matched pairs of noisy images (25,26). The requirement for any matched images is fully removed in the Noise2Void (n2v) technique, where learning and removal of noise are carried out based on a single noisy image data set (26, 27). Reconstruction based on a single noisy data set also allows per-image training, thus compensating for day-to-day variability of, for example, fluorescence labeling or fine-adjustment of optical parts.

A second obstacle to the wide application of deep learning methods is the possibility of errors in the reconstructed fluorescence images (23,24). These errors manifest as deviations between the high-quality ground truth images and the images reconstructed from low SNR data. A dilemma arises, where the effective application of deep learning networks can only proceed without acquisition of ground truth data, but ground truth data are required to assure the experimenter that reconstruction is error-free. In this work, we develop a pragmatic work-flow for the quality-controlled adjustment and application of n2v for denoising in high-speed fluorescence microscopy. In this work-flow, for every acquired view of a given sample, a small data set with high-quality data is recorded to control reconstruction quality, followed by full time-lapse acquisition of only compromised data. We demonstrate the applicability of this work-flow in the analysis of fluctuations in molecular clusters in live zebrafish embryos. Our analysis reveals a close coordination between post-translational modifications of RNA polymerase II (Pol II) and changes in the 3D shape of these clusters on the scale of a few seconds. These observations are confirmed by an alternative experimental approach, where still images from chemically fixed cells are sorted based on an additional fluorescence marker for genes that transiently engage with the molecular clusters. Our approach provides a guideline for other microscopists interested in the quality-controlled application of ground-truth-free image reconstruction methods. The approach can be implemented on fluorescence microscopes with typical specifications used for time-lapse recordings without the need of software development or hardware control beyond the standard functionality.

## Results

### Quantification of image reliability and effective resolution in reconstructed microscopy images

**Fig. 1.**
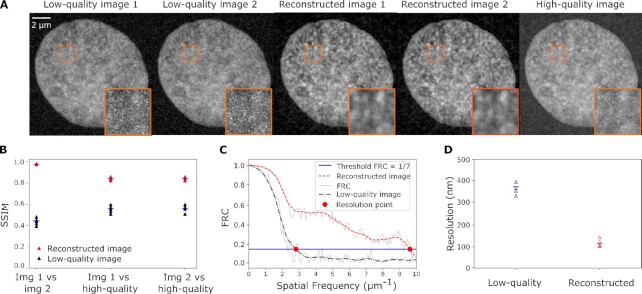
Metrics for the reliability and effective resolution in n2v-reconstructed images. (A) Representative micrographs of the DNA distribution in a nucleus in a fixed zebrafish embryo, recorded with a stimulated emission depletion (STED) super-resolution microscope. The same image plane was recorded twice at low quality, once at high quality, and two n2v-reconstructed images were prepared from the low-quality images. (B) SSIM values for pair-wise comparison (image 1 vs. image 2) and comparison against the high-quality image (image 1 vs. high-quality and image 2 vs. high-quality) for the low-quality images and the reconstructed images. (C) FRC curves calculated based on a low-quality image pair and the corresponding reconstructed image pair. (D) FRC-based effective resolution for four pairs of low-quality images and the corresponding pairs of reconstructed images.

The structural reliability and effective spatial resolution of reconstructed images can be assessed by a combination of widely used metrics. The structural reliability can be assessed via the structural similarity index metric (SSIM). SSIM quantifies the similarity between two images and returns a value between 0 and 1 (28,29). SSIM values close to 1 indicate that two images are very similar, lower SSIM values indicate images that are less similar. One application is the comparison of two images obtained with the same acquisition and postprocessing steps, providing a quantification of the reliability of the obtained images. Using SSIM, we can, for example, demonstrate how changes in image acquisition settings, such as the reduction of exposure time, can compromise image reliability (Fig. S1, Supplementary Material). Applying n2v to pairs of super-resolution microscopy images, we can illustrate how denoising can increase image reliability (Fig. 1A and B). SSIM can also be used to assess whether reconstructions of low-quality images obtained with, for example, low exposure times can approximate high-quality images (Fig. 1A and B). The assessment of image reliability via SSIM is, however, not sensitive to localized differences between images, as are typically introduced at edges during denoising procedures. Such local occurrences of unreliable reconstruction are readily detected by the local SSIM (Fig. S2, Supplementary Material) (30). The combination of SSIM and local SSIM, thus, allows an assessment of image reliability based on paired images, as well as the similarity between a reconstructed and a corresponding high-quality image.

A key aspect of performance in microscopy is the effective image resolution. The effective image resolution is determined by both the optical resolution of a given imaging instrument, and by the ratio of photons emitted by the structure of interest over polluting photons, often referred to as SNR. This effective resolution can be quantified via Fourier ring correlation (FRC) (31,32). FRC evaluates the similarity of a pair of images in frequency space, so as to determine the spatial frequency up to which the images are consistent with each other (Fig. 1C). The inverse of this spatial frequency is then taken as the effective spatial resolution (Fig. 1C). Applying the FRC metric to our super-resolution microscopy data reveals that, indeed, n2v-denoising can recover effective resolution in low-quality images (Fig. 1D; Fig. S3, Supplementary Material). Taken together, SSIM and FRC can objectively assess image reliability and effective resolution in matched pairs of reconstructed images.

### Optimization of exposure time for high-speed time-lapse imaging

**Fig. 2.**
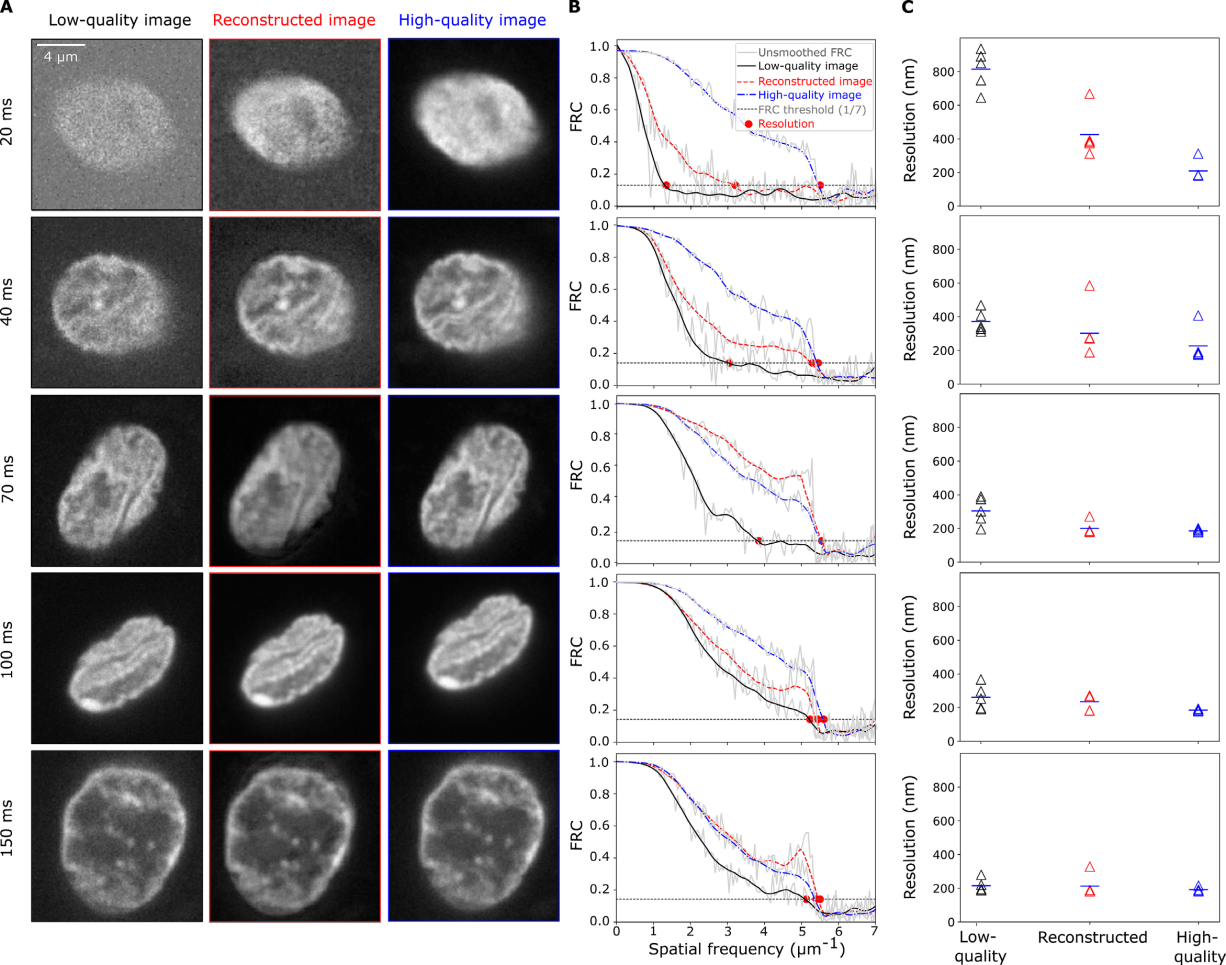
Metric-based estimation of how far image quality can be compromised while still allowing recovery of effective resolution by denoising. (A) Representative micrographs of nuclei of human cheek cells for different camera exposure times (*t_exp_*, as indicated), all high-quality images were acquired at the same position but with an exposure time of 200 ms. Images are maximum-intensity projections, DNA was labeled by Hoechst 33342. (B) FRC curves calculated from a pair of matched low-quality images, from a pair of reconstructed images, and a pair of high-quality images for the different *t_exp_*. (C) Effective resolution for the indicated *t_exp_*, *n* = 5 nuclei per *t_exp_*, values are shown with mean.

While denoising with n2v can, in principle, reconstruct images acquired with reduced exposure time (*t_exp_*), for a given experiment it is not known a priori just how far *t_exp_* can be reduced while ensuring a sufficient image reconstruction. To demonstrate how SSIM and FRC can guide the choice of *t_exp_*, we carried out live sample microscopy of cells obtained from buccal smears (“human cheek cells”) for a range of exposure times, $t_{exp}= 20, 40, 70, 100, 150\,\text{ }\mathrm{ms}$ (Fig. 2A). For each *t_exp_*, a n2v-network was separately trained on a pair of images and the effective resolution for these reconstructed images was assessed (Fig. 2B). For $t_{exp}=70\,\, \mathrm{ms}$ or higher, an effective resolution of $\sim 200\,\, \mathrm{nm}$ was attained for the reconstructed images (Fig. 2C). This resolution was not further improved by longer exposure times, but could not be attained for shorter exposure times (Fig. 2C). This FRC-based assessment suggests $t_{exp}=70\, \mathrm{ms}$ as an optimal exposure time. We controlled the structural reliability of the reconstructed images by local SSIM, finding reconstruction errors for $t_{exp}=20\, \mathrm{ms}$ (Fig. S2A–G, Supplementary Material). Considering both the FRC and local SSIM results, all $t_{exp}\ge 40\,\, \mathrm{ms}$ seem structurally reliable, while only $t_{exp}\ge 70\,\, \mathrm{ms}$ allow maximal effective image resolution after image reconstruction. In this setting, the experimenter can, therefore, choose between faster acquisition ($t_{exp}= 40\,\, \mathrm{ms}$) or higher effective resolution ($t_{exp}= 70\,\, \mathrm{ms}$), all while ensuring a high certainty of structural reliability.

### A two-phase acquisition protocol for quality-controlled denoising of time-lapse recordings

**Fig. 3.**
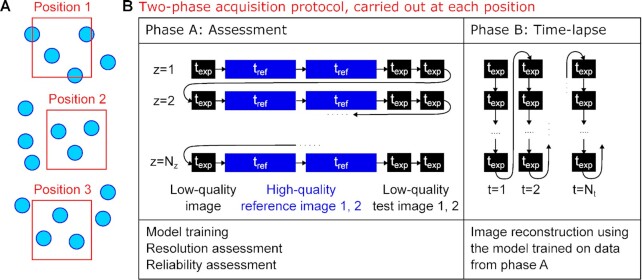
A two-phase acquisition protocol to combine acquisition of quality control images with high-speed time-lapse imaging. (A) Image data were acquired at multiple positions in a sample, thus obtaining multiple viewpoints containing several objects of interest (nuclei, indicated as circles). (B) For each position, a sequence of two acquisition phases is carried out. In phase A, for each z position, a low-quality image, two high-quality reference images, and two low-quality test images are recorded. Low-quality images are recorded at a shortened exposure time (*t_exp_*), high-quality images at a reference exposure time resulting in images of the desired quality (*t_ref_*). Acquisition phase A obtains the images required for n2v model training as well as the assessment of effective image resolution and reconstruction errors. In phase B, only single low-quality images are recorded with the shortened exposure time (*t_exp_*), resulting in an increased rate of acquisition compared to acquisition with full exposure time (*t_ref_*). Acquisition phase B obtains only low-quality images, which are reconstructed after the experiment is completed.

To integrate the metric-based assessment of n2v-processed images with the recording of high-speed time-lapse data, we propose an acquisition protocol that contains two distinct phases and is carried out at every position in a given sample (Fig. 3A). In the first phase (A, assessment), all image data required for the application of SSIM and FRC metrics are recorded (Fig. 3B). In particular, for each of the image planes that make up the acquired 3D volume, the following images are obtained: one low-quality image (*t_exp_*), two high-quality images recorded with the longer reference exposure time (*t_ref_*), followed by two more low-quality test images (*t_exp_*). In the second phase (B, time-lapse), a sequence of 3D volumes is acquired with only a single low-quality image for each of the image planes, reducing the time spent for the acquisition of a 3D volume (Fig. 3B).

The data acquired by this two-phase acquisition protocol allow a comprehensive quality control assessment for every recorded position. Specifically, we first train a n2v-network for each position, with which we reconstruct the low-quality test images 1 and 2. We can then assess the effective resolution using the FRC metric and additionally control the reconstructed image for reconstruction errors using SSIM and local SSIM. For positions where a sufficient effective resolution is achieved by the reconstruction, and a sufficiently low level of reconstruction error is found, the trained n2v-network is then applied to the time-lapse data from phase B, thus providing n2v-reconstructed time-lapses with per-position quality control.

### High-speed imaging reveals coordinated changes of phosphorylation and shape of Pol II clusters

To demonstrate the applicability of our proposed protocol for quality-controlled n2v-supported live imaging protocol, we attempted to visualize changes in the molecular state as well as the 3D shape of macromolecular clusters enriched in Pol II. To this end, we recorded microscopy images from live zebrafish embryos with an instant-SIM microscope (33). We visualized Pol II that is recruited to macromolecular clusters (Pol II Ser5P) or has transitioned toward production of RNA transcripts (Pol II Ser2P) by fluorescently labeled antibody fragments (Fabs). These Fabs have been validated to specifically and reliably detect changes in the Pol II Ser5P and Pol II Ser2P levels in zebrafish embryos, and do not perturb embryonic development in any obvious fashion (34–36). To establish exposure times, we first adjusted imaging parameters so as to obtain images that reveal cluster shape in the Pol II Ser5P channel on the microscope’s live display without any processing. We chose this reference exposure time as $t_{ref}=200\,\, \mathrm{ms}$, resulting in an overall time of $6\,\, \mathrm{s}$ that is required to obtain a full 3D image stack. Using $t_{ref}=200\,\, \mathrm{ms}$, we recorded image data in line with the two-phase acquisition protocol, with the phase B spanning a total time of 2 min. Specifically, we recorded data for four different exposure times ($t_{exp}=10,20,50,100\,\, \mathrm{ms}$; Fig. S4A–C and Table S1, Supplementary Material). For all *t_exp_*, we achieve an effective resolution of 400 nm (lateral) or better after n2v-based reconstruction, which compares favorably to an effective resolution of approximately 700 nm in the high-quality images (Fig. S4D, Supplementary Material). A comparison against conventional, state-of-the-art 3D deconvolution also indicates a stronger improvement of effective image resolution by n2v (Fig. S5, Supplementary Material). Analysis by local SSIM suggests that reconstructions for $t_{exp}\ge 20\,\,\mathrm{ms}$ offer a reliability similar to a comparison between two high-quality images, reconstructions of images obtained with $t_{exp}=10\, \mathrm{ms}$ are prone to reconstruction errors (Fig. S4E, Supplementary Material). Accordingly, we selected images acquired with $t_{exp}=20\, \mathrm{ms}$ (effective lateral resolution $\sim 400 \,\, \mathrm{nm}$) and $t_{exp}=50\, \mathrm{ms}$ (effective lateral resolution $\sim 350 \, \mathrm{nm}$) for further analysis, which provided full 3D image stacks at a time resolution of $1\, \mathrm{s}$ and $2\, \mathrm{s}$, respectively.

**Fig. 4.**
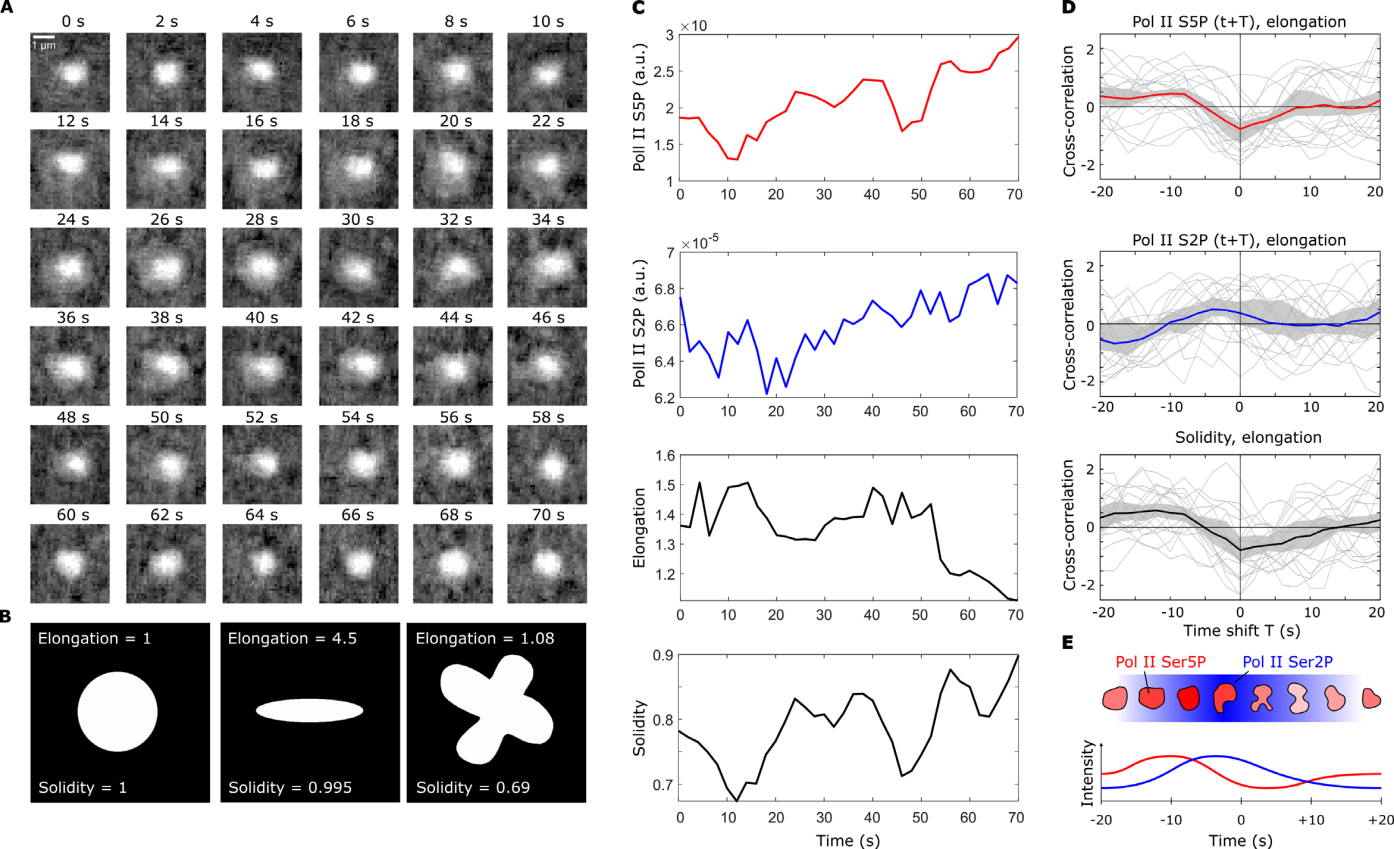
n2v-accelerated imaging reveals coordinated changes in shape and phosphorylation levels of Pol II clusters on the scale of seconds. (A) Representative series of time-lapse images showing a single Pol II cluster in the Pol II Ser5P channel (single image plane from the middle z position of the cluster, exposure time $t_{exp}=50\, \mathrm{ms}$, effective time resolution for full 3D volume acquisition of $2\, \mathrm{s}$). The Pol II Ser2P channel is not shown because only average intensity, not shape was quantified from this channel. (B) Example shapes to illustrate how elongation and solidity represent object shape. (C) Time courses of Pol II Ser5P intensity, Pol II Ser2P intensity, elongation, and solidity for the example time-lapse shown in panel A. (D) Cross-correlation analysis of the temporal coordination of Pol II Ser5P intensity, Pol II Ser2P intensity, and solidity with elongation. Gray lines indicate the time-shifted correlation for single cluster time courses, thick lines indicate the mean, and the gray region the 95% bootstrap CI. Analysis based on *n* = 30 tracked clusters, recorded from one sphere stage embryo. (E) Summary of the coordinated changes in phosphorylation and cluster shape suggested by the cross-correlation analysis. A stereotypical sequence of events can be seen: cluster Pol II Ser5P intensity transiently increases (red) and the cluster becomes rounder, then cluster Pol II Ser2P transiently intensity increases (blue), until finally the cluster transiently unfolds and becomes elongated.

As previously observed, clusters seen in the Pol II Ser5P channel were persistent during the entire phase B acquisition period (36). The n2v-processed Pol II Ser5P time-lapse images were segmented to detect Pol II-enriched clusters, each cluster was then tracked over the whole time-lapse based on spatial proximity in consecutive time points (Fig. 4A). Based on the Pol II Ser5P-derived segmentation masks, Pol II Ser5P and Ser2P intensities as well as shape quantifiers could be determined for each time point (Fig. 4B). We used the two shape quantifiers elongation, which indicates how much an object is stretched along its main axis, and solidity, which indicates how convex (“rounded out”) the outline of a given shape is. The resulting time courses exhibit fluctuations, and the question arises whether a systematic relationship exists between the different quantities (Fig. 4C). Indeed, a cross-correlation analysis that was anchored on cluster elongation suggests a systematic relationship (Fig. 4D). The cross-correlation analysis reveals an initial increase in Pol II Ser5P intensity, followed by a transient increase in Pol II Ser2P intensity ∼5 s later, and a transient decrease in Pol II Ser5P intensity another ∼5 s later. These changes are accompanied by an initial rounding up of clusters (solidity increase), followed by transient unfolding (solidity decrease) ∼10 s later. These cross-correlation analysis results are obtained at both $t_{exp}=50\, \mathrm{ms}$ (Fig. 4) and $t_{exp}=20\, \mathrm{ms}$ (Fig. S6, Supplementary Material), indicating that our findings are not mere coincidence. Our observations are representative of a stereotypical sequence of events, which occurs repeatedly and is, therefore, detected by the cross-correlation analysis: Pol II Ser5P intensity increases and the cluster rounds up via the rapid recruitment of Pol II to a given cluster, Pol II Ser5P intensity decreases and Pol II Ser2P intensity increases as some of the recruited Pol II proceeds into transcript production, while the cluster gets elongated and unfolded (Fig. 4E). Previous work indicates that transcribing Pol II and the resulting nascent RNA transcripts induce distinct rearrangements in molecular clusters, providing a potential cause for the elongation and unfolding (35–38). Notably, these works suggest that changes in Pol II state and cluster organization come about due to transient engagement of genes with Pol II-enriched clusters, leading to the induction of genes and to their release from these clusters.

### Pseudo-time analysis from fixed sample images also detects coordinated changes in phosphorylation and cluster shape

**Fig. 5.**
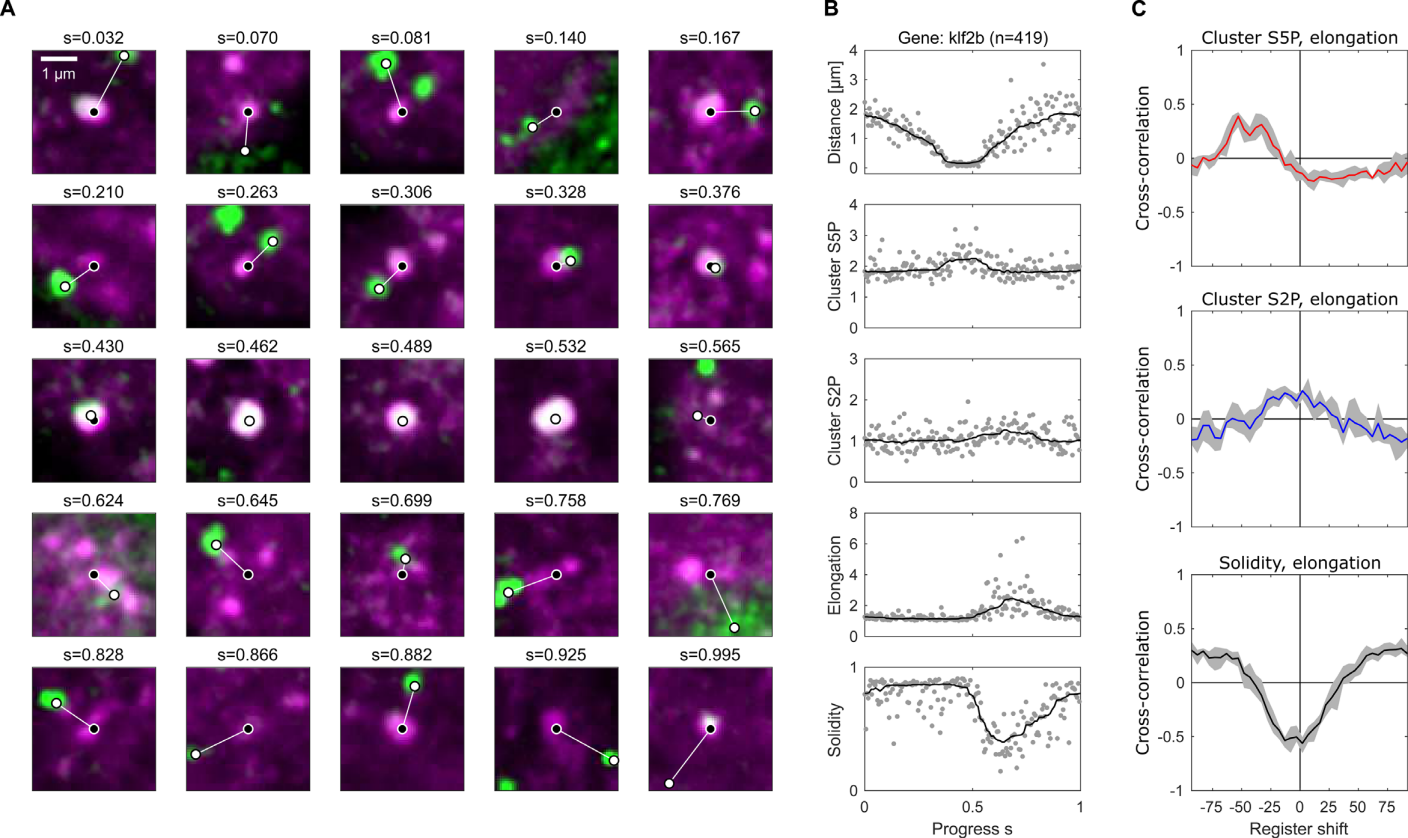
Pseudo-time analysis of data from fixed embryos relates transient engagement and activation of a gene to the phosphorylation and shape changes observed in live embryos. (A) Example images of Pol II Ser5P (magenta signal) clusters sorted by a pseudo-time progress coordinate (*s*, periodic, defined on the interval [0,1)), which is calculated based on interaction with the gene *klf2b* (green represents oligopaint fluorescence in situ hybridization signal for *klf2b*). Center positions (weighted centroid) are indicated for the Pol II Ser5P cluster (white circle with black filling) and the gene (black circle with white filling) and connected with a white line for illustration. For details of the reconstruction, see Figure S8 (Supplementary Material). For an overview containing all eight genes that were assessed, see Fig. S9B (Supplementary Material). (B) Pol II Ser5P and Ser2P intensity, elongation, and solidity of Pol II Ser5P clusters sorted by pseudo-time *s*. A total of *n* = 186 clusters from *N* = 4 independent samples, obtained in two independent experiments, were included in the analysis. (C) Cross-correlation analyses for different register shifts in the coordinate *s*, the register shift is in units of data points by which the coordinate *s* was shifted. Gray regions indicate 95% bootstrap CI.

To verify the conclusions obtained by the fluctuation analysis, we assessed changes in cluster state by an independent approach based on the interaction with a gene. Specifically, we fixed zebrafish embryos in the sphere stage, and fluorescently labeled a panel of eight genes as well as Pol II Ser5P and Pol II Ser2P (Fig. 5A; Tables S2–S4, Supplementary Material). In most nuclei, zero to four labeled foci representing the labeled genes could be detected, in line with the expected counts for two alleles that undergo replication before cell division (Fig. S7, Supplementary Material). Fixation of samples prevents live imaging, thus removing the temporal information from the images. In exchange, images with distinctly higher signal can be obtained without the need of n2v-processing, and the location of the labeled gene can be used as additional information that is not available in our live imaging data. The analysis of the obtained image data was, therefore, based on gene-Pol II cluster interaction pairs. An interaction pair is constructed by the detection of the location of a labeled gene, and by logical association of this gene with the Pol II Ser5P cluster that is closest in space (Fig. S8A and B, Supplementary Material). For each interaction pair, fluorescence intensities of the gene, fluorescence intensities of the Pol II cluster, distance between both objects, and shape properties of the Pol II cluster were combined into a vector representing the interaction pair. Principal component analysis of these pairs revealed a cyclical pattern, based on which a pseudo-time coordinate was constructed (Fig. 5B; Fig. S8C, Supplementary Material). In particular, the two first principal component coordinates of each pair were transformed into an angle coordinate using the 2-argument arctangent (atan2) function. This angle coordinate was divided by 2π to obtain a pseudo-time coordinate *s* in the range from 0 to 1. The assignment of a pseudo-temporal order to image data obtained from fixed samples has been used previously, for example for the nanoscale assessment of endocytosis (39, 40). Ordering the interaction pairs along the pseudo-time coordinate allowed the extraction of time-shifted correlations (Fig. 5C), which directly mirrored those we obtained from our live imaging data (Fig. 4D). We suspected that the location of a gene that interacts with Pol II Ser5P clusters provides the crucial information for successful pseudo-time reconstruction (genes *foxd5, klf2b*, and *zgc:64022*; Fig. S9, Supplementary Material). Indeed, when we attempted pseudo-time reconstruction on the full panel consisting of eight genes, we found that for genes that only rarely come close to Pol II Ser5P clusters, the pseudo-time approach failed to reproduce the correlation analysis results (genes *vamp2, ripply1, drll.2, gadd45ga*, and *iscub*; Fig. S9, Supplementary Material). In the case of successful pseudo-time reconstruction, our results suggests that a gene visits a Pol II Ser5P cluster in close coordination with changes that occur in the Pol II cluster. Specifically, genes engage in close contact when cluster Pol II Ser5P intensity increases, and detach at a time when clusters undergo transient elongation (genes *foxd5, klf2b*, and *zgc:64022*; Fig. 5A and B; Fig. S9, Supplementary Material). The time-scales of this interaction can be estimated by a comparison of the distance between the cross-correlation maximum and minimum in the cluster Pol II Ser5P signal (∼50 steps in pseudo-time, corresponding to $\sim 10\, \mathrm{s}$ in the cross-correlation analysis based on live-imaging results) and the total number of observed interaction pairs (169, 186, and 191 for *foxd5, klf2b*, and *zgc:64022*, respectively), implying an average duration of $\sim 36\, \mathrm{s}$ between two consecutive interaction events. To conclude, the correlation analysis based on pseudo-time reconstruction provides an independent confirmation of the coordination between Pol II phosphorylation levels and cluster shape obtained by n2v-supported live imaging. This agreement suggests that these two approaches provide complementary views of the same, stereotyped sequence of changes in molecular properties and the shape of Pol II clusters.

## Discussion

**Fig. 6.**
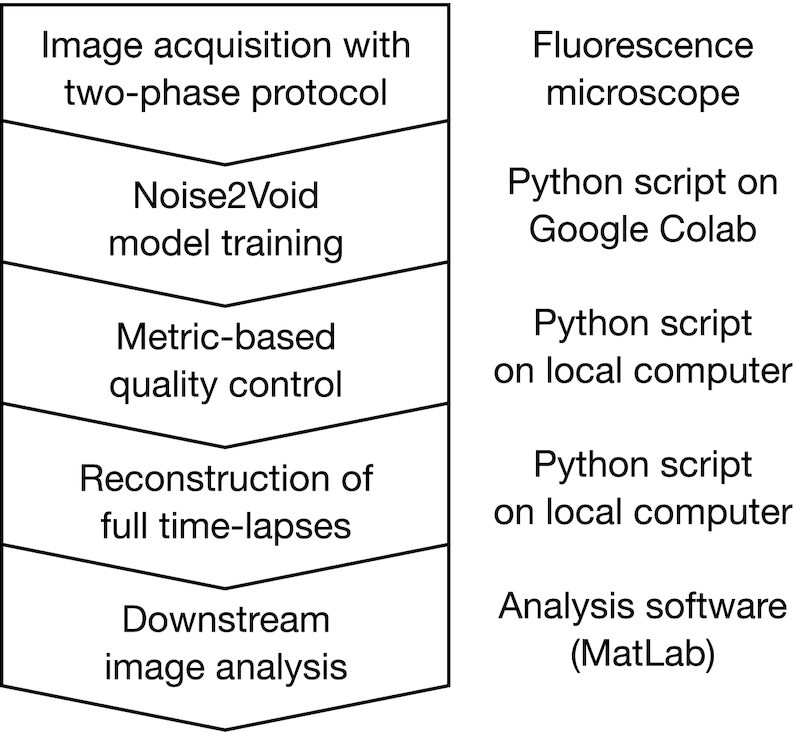
Work-flow for n2v reconstruction for time-lapse data with quality control.

In this study, we describe how the quality of images that are reconstructed by deep-learning algorithms can be controlled for, addressing the specific case of unsupervised denoising by n2v (for an overview of the work-flow, see Fig. 6). We implemented our approach of quality control toward the acceleration of high-speed imaging, where camera exposure times are reduced and the resulting loss of signal quality is recovered by n2v-denoising. We then apply our approach to the example of imaging the molecular state and the shape of Pol II clusters in live zebrafish embryos. Our work illustrates how, in a practical application setting, the performance improvements from deep-learning algorithms in fluorescence microscopy can be combined with a high level of confidence in the reconstructed images. The tools used in our study are designed for data sets consisting of ordered 2D images (“hyper-stacks”), limiting their immediate application to these types of image data.

We specifically apply our quality control approach to an unsupervised denoising technique, the deep learning-based tool n2v (27). Currently, reconstructions that map from noisy to high-quality data on the basis of paired training image data offer the highest reconstruction performance (22). In many practical settings, such pairs of noisy and high-quality images cannot be obtained. An alternative is offered by reconstructions based on matched pairs composed of noisy images only (n2n) (26). Further developments now offer the possibility to reconstruct high-quality images directly from single noisy images (Noise2Self (41) and n2v (27)). Such a self-supervised approach seems ideally suited to reconstruction tasks where fluorescence labeling exhibits strong variability, optical components are changed between different experiments, or sample properties vary on a day-to-day basis. These characteristics are typical of biological microscopy applications, highlighting the applicability of self-supervised reconstruction methods in this area. A crucial assumption of self-supervised denoising approaches is that the noise in each pixel is an uncorrelated sample from the same probability distribution. Newer variants of these algorithms explicitly adjust the probability distribution of the noise to different parts of the image, thus improving the results where additional information on the noise characteristics is available (19,42, 43). Yet, other variants model the structure of the signal itself (44). These newer variants of self-supervised denoising could provide further improvements in reconstruction performance, while retaining most of the pragmatic applicability of self-supervised reconstruction methods.

We base our assessment of image quality on two metrics, (local) SSIM and FRC. More generally, metrics for image quality assessment belong to three main groups of functionality. The first group includes methods assessing the quality of images against a corresponding reference image (high-quality image). These methods are called full-reference, emphasizing the need for high-quality reference data (28,45). SSIM and consecutive similarity (CSS) metric, which is a variation of SSIM (46), are in this category. We used (local) SSIM, which provides an error map by structurally comparing the reconstructed image with the reference image, and based on that error map controlled for reconstruction defects. The second group, called reduced-reference, contains methods which are not using matched reference images, but rather general knowledge of properties and statistics that are typical of a set of reference images (47). Natural scene statistics (NSS) is one major method in this category (45). The underlying hypothesis of all NSS-based method is that all the original images are “natural” and that a distortion process introduces some unnaturalness that can be quantified by deviation from models of natural signals. Due to the day-to-day variability of the signals produced by fluorescence microscopy of biological samples, modeling natural signals appears challenging. The third category of image quality assessment methods is called no-reference, because quality assessment proceeds without a matched reference image or other prior knowledge (48). FRC is in this category and we used it to assess the spatial resolution of the reconstructed images. Based on the achieved spatial resolution, we could decide how far exposure times could be reduced while still supporting successful denoising. One tool that implements several of these metrics for the assessment of local anomalies in super-resolution microscopy data is SQUIRREL (49). The quality scores and error mapping provided by SQUIRREL can, in principle, also be applied to images reconstructed by deep-learning methods.

The image acquisition protocol we propose consists of a phase during which all necessary data for quality control are collected for a single time point (phase A), followed by high-speed time-lapse imaging with compromised image quality (phase B). This protocol seems appropriate for the acquisition of short bursts of images, where the main limitation lies in how many images can be acquired in a short amount of time. For other imaging challenges, different protocols could be developed. In a different situation where, for example, photo-bleaching limits the acquisition of long time courses, excitation light levels could be reduced, and the compromised signal could be recovered by denoising. In such an experiment, quality control points could be placed at regular intervals over the course of acquisition. In a setting where, for example, sample structure or the level of fluorescence labeling changes significantly over the course of recording, a quality control phase at the beginning and at the end of the experiment might be advisable. Besides the implementation of additional control points in the experimental procedure, such extensions of our simple two-phase protocol would need no further modification to the quality control approach we used in our work.

Our live-sample microscopy recordings reveal a stereotypical sequence of events, where the Pol II recruitment and pause–release steps of transcriptional induction are closely coordinated with changes in the shape of Pol II clusters. While previous studies achieve high spatial or temporal resolution, our approach combines high resolution in time as well as in space. Our temporal resolution of 1 to 2 s for a full 3D stack is comparable to previous assessments of Pol II localization (50,51). These studies, however, do not monitor the specific phosphorylation states associated with Pol II regulation. Imaging of these phosphorylation states was previously performed with an effective time resolution of 1 min for a single gene (38) or 10 s for an engineered gene array (52) for the acquisition of full 3D volumes. By fitting of kinetic models of Pol II regulation, these studies suggest rates of pause release of 2 to 2.5 min and production of the first 1 kb of transcript length within 2.5 min (assuming an elongation rate of 0.4 kb per min) (38, 52). Photobleaching experiments assessing endogenous Pol II combined with computational modeling indicated 2.3 s for initiation and 42 s of pausing at the promoter, as well as an elongation rate of 2 kb per min (53). These estimates for the elongation rates fit well with estimates of approximately 1 kb per min from alternative sequencing-based approaches (54). Lastly, another study suggests that 6.3 s are sufficient for Pol II to loosely associate with an induced gene as well as proceed into elongation (50). While these estimates for the duration of induction and pause–release imply a broad spectrum of kinetics, our estimates of 2 to 3 s for pause release and approximately 36 s for the duration of one complete gene–cluster interaction cycle fall within the previously estimated range for pause release and RNA production. Besides temporal coordination, also relative distances have been assessed, for example between Pol II clusters and nascent mRNA (38) and between enhancers, Pol II, and the transcription start site (55,56). In these studies, nascent mRNA is displaced 100 to several hundred nm relative to sites harboring transcriptional regulators and recruited Pol II. This displacement is in line with our observations that genes that undergo elongation are located outside of Pol II Ser5P clusters. In contrast to previous work, our approach reveals the full shape of the Pol II Ser5P clusters. Taken together, the kinetics of single-gene induction suggested by our live-sample experiments seem in line with previous work, and the spatial organization of clusters and interacting genes directly correlates with previous work assessing relative distances of different components of the transcriptional machinery.

Our pseudo-time reconstruction revealed that the changes in Pol II phosphorylation and cluster shapes are temporally coordinated with the visit of genes to the Pol II clusters. Previous work suggests that the Pol II clusters in early embryonic development form on regulatory chromatin regions, including superenhancers (36,57, 58). Accordingly, our data seem to directly show single genes that undergo transcriptional activation during a visit to Pol II-enriched clusters that contain regulatory chromatin regions. Different models for such enhancer–promoter communication in transcriptional control were proposed (59–61). The stereotypical sequence suggested by our data fits most closely to a condensate hit-and-run model, where genes transiently interact with enhancer-associated condensates for transcription initiation, and leave from the condensate in association with the onset of transcriptional elongation (61). A condensate hit-and-run model can also explain earlier observations suggesting cyclic interactions, where genes repeatedly engage with and depart from Pol II-enriched clusters (62). Such a model also could support the proximity-dependent activation of *Shh* by its enhancer *ZRS* (63,64). The activation of genes by enhancers was also found to not require direct contact, but can occur over a distance of 200 nm or more (65,66). These observations, together with evidence in support of the condensate hit-and-run model, allow speculations about a liquid-bridge model of enhancer–gene communication. In such a liquid-bridge model, genes transiently become embedded within an enhancer-associated condensate, allowing the transfer of transcriptional machinery, including Pol II, to the gene promoter (61, 67,68). While previous work indicates that the onset of RNA production at newly activated genes results in their exclusion from the enhancer-associated condensates (37,69–72), the initial engagement with the enhancer-associated condensates is less well understood. Such an engagement would, however, be naturally explained by the formation of small condensates at promoters. Such condensates could emerge, for example, at CpG-rich regions that are placed directly upstream of promoter regions of many developmental genes and were found to contribute to gene–promoter contacts in 3D space (73).

## Methods Summary

### Live imaging of primary cell culture of human cheek cells

Short-term primary cell cultures of human cheek cells were obtained by a buccal smear, stained with Hoechst 33342, and transferred into an 8-well ibidi μ-Slide (#1.5 selected glass) for microscopy. Microscopy data were recorded using a commercial implementation of the instant-SIM high-speed super-resolution confocal microscopy principle (VisiTech iSIM) (33) built on a Nikon Ti2-E stand. A Nikon Silicone Immersion Objective (NA 1.35, CFI SR HP Plan Apochromat Lambda S 100XC Sil) was used. Detailed description see Supplementary Material Appendix.

### Zebrafish husbandry, live imaging, and fixation

Embryos were obtained through spontaneous mating and dechorionated with pronase. For STED microscopy of DNA in fixed zebrafish embryos protocols from our previous work were followed (74). STED microscopy was performed using a Leica TCS SP8 STED microscope with a 775-nm depletion line and a motorized-correction 93x NA 1.30 glycerol objective (HC PL APO 93X/1.30 GLYC motCORR). For live-imaging of Pol II CTD phosphorylation, embryos were microinjected with covalently labeled fragments of antibodies (Fab). Microscopy images were recorded by iSIM with the silicon immersion objective. Detailed description see Supplementary Material Appendix.

### Oligopaint FISH and immunofluorescence

Following protocols from our previous work (36), fixed sphere-stage zebrafish embryos were subjected to oligopaint FISH labeling of genomic regions surrounding zygotically expressed genes (75–78), followed by indirect immunofluorescence detection of Pol II CTD phosphorylation, and mounted in Vectashield H-1000 under #1.5 selected cover glass. Image data were recorded by iSIM using a Nikon Oil Immersion Objective (NA 1.49, CFI SR HP Apo TIRF 100XAC Oil). Detailed description see Supplementary Material Appendix.

### Metrics for image assessment

For SSIM and local SSIM analysis, we only used the structural term

$$\begin{equation*}
SSIM(x,y)=\frac{2\sigma _{x,y}+C_3}{\sigma _ x\sigma _y+C_3}.
\end{equation*}
$$

The FRC analysis is based on the cross-correlation of two images in frequency space:

$$\begin{equation*}
FRC(r)=\frac{\sum _{r_i\in r} F_1(r_i)\cdot F_2(r_i)^{*}}{\sqrt{\sum _{r_i\in r} (F_1(r_i))^2\cdot \sum _{r_i\in r}(F_2(r_i))^2}},
\end{equation*}
$$

where *F*_1_, *F*_2_ are the Fourier transforms of two images and *r_i_* refers to all frequency space bins that fall within a given ring radius *r*. Images are considered reliably resolved up to the frequency *F*(*r*) for which *FRC*(*r*) falls below the commonly used threshold value of 1/7. Details are described in Supplementary Material Appendix.

### Image analysis

The analysis of Pol II clusters in live-imaging data as well as the pseudo-time reconstruction from fixed-sample data were implemented in the form of MatLab scripts. The analysis steps and script availability are described in SI Appendix.

## Authors’ Contributions

Designed research: H. H., I. M., R.P., A.P., A.K., L.H. Performed research: H.H., I.M., R.P., A.P., L.H. Analyzed data: H.H., R.P., L.H. Wrote the paper: H.H., I.M., A.K., L.H.

## Supplementary Material

pgac065_Supplemental_Files

## Data Availability

All raw data and analysis code are provided as supplementary materials or via publicly accessible repositories. All links to repositories are listed in the supplementary material. The raw data obtained from human cheek cells cannot be shared due to ethical restrictions.
