# Supplementary material for: Deep-learning microscopy image reconstruction with quality control reveals second-scale rearrangements in RNA polymerase II clusters
Source: PNAS Nexus. 2022 May 23;1(3):pgac065. doi: 10.1093/pnasnexus/pgac065 (PMC9896941; doi:10.1093/pnasnexus/pgac065)
Supplement: pgac065_Supplemental_Files [file pgac065_supplemental_files.zip › PNASNEXUS-PNASNEXUS-2022-00064-T-s08.pdf]

Supplementary Materials

Scripts used for the selection of genes to be labeled by oligopaint FISH:

`Oligopaint_GeneSelection_ProbeDesign_Scripts.zip`

Text file used to order the oligo pool:

`OligopaintsToOrder_Oct2020.txt`

Materials and Methods

Live imaging of primary cell culture of human cheek cells

Cells were obtained by a buccal smear with a P1000 pipette tip. Short-term primary cell cultures were then created by adding the pipette tip to a micropipette and pipetting up and down several times in 2 ml of PBS (Dulbecco's formulation) with 0.8 mM $CaCl_2$ and 4 μM Hoechst 33342. 500 μl of this primary cell culture were transferred to one well of an 8-well ibidi μ-Slide (8-well glass bottom #1.5 selected D263 M Schott glass). The ibidi slide was sealed with parafilm to prevent evaporation and incubated for 1 h at room temperature to ensure flattening of the cell nuclei before microscopy. Participants provided free and informed consent. Procedures were reviewed and accepted by the Karlsruhe Institute of Technology ethics committee. Raw image data were stored in an anonymous fashion and are not for public release.

Zebrafish husbandry

All zebrafish husbandry was performed in accordance with the EU directive 2010/63/EU and German animal protection standards (Tierschutzgesetz §11, Abs. 1, No. 1) and is under supervision of the government of Baden-Württemberg, Regierungspräsidium Karlsruhe, Germany (Aktenzeichen35-9185.64/BH KIT). Embryos used for the different experiments were obtained through spontaneous mating of adult zebrafish. Collected embryos were dechorionated with pronase, washed 3 times with E3 embryo medium, once with 0.3x Danieau's solution, and subsequently kept in agarose-coated Petri dishes or 6-well plates in 0.3x Danieau's solution at 28.5^{o}C.

STED microscopy of DNA in fixed zebrafish embryos

Following protocols from our previous work [74], sphere-stage zebrafish embryos were fixed overnight in 0.3x Danieu's solution supplemented with 2% formaldehyde and 0.2% Tween-20 at 4°C, permeabilized for 15 min using 0.5 % Triton X-100 in PBS, washed three times with PBS supplemented with 0.1% Tween-20, and mounted in TDE-media supplemented with 10x SPY-595 DNA fluorescence stain under selected #1.5 glass cover slips. STED microscopy was performed using a Leica TCS SP8 STED microscope (Leica Microsystems, Wetzlar, Germany) with a 775 nm depletion line and a motorized-correction 93x NA 1.30 glycerol objective (HC PL APO 93X/1.30 GLYC motCORR). 100% 3D STED depletion was used.

Live imaging of RNA Pol II CTD phosphorylation in zebrafish embryos

One-cell-stage embryos were dechorionated with pronase in 0.3x Danieau's solution and covalently labeled fragments of antibodies (Fab) were micro-injected into the yolk. In each embryo, 1 nl of Fab mix (0.2 μl 1 % Phenol Red, 1.5 μl A488-labelled anti-Pol II Ser2P Fab, 3.3 μl JF646-labelled anti-Pol II Ser5P Fab, Fab stock cencentration approximately 1 mg/ml) was injected. The embryos were mounted for microscopy in 0.7% low-melting agarose in 0.3x Danieau's solution in ibidi 35 mm imaging dishes (#1.5 selected glass cover slips) at 512-cell stage of development, images were acquired at the sphere stage.

Oligopaint FISH and immunofluorescence in fixed whole embryos

Embryos were fixed in the sphere stage of development (4% formaldehyde, 0.2% Tween-20 in 0.3x Danieau's, fixation overnight at 4°C), permeabilized (0.5% Triton X-100 in PBS, 15 min), washed in PBS with 0.1% Tween-20 (PBST) for 2 minutes, and treated with 0.1 N HCl for 5 min. A sequence of wash steps followed: twice with 1 ml 2x saline sodium citrate buffer with 1% Tween-20 (2xSCCT), once with 2xSCCT+50% formamide for 2 min at room temperature, and once with preheated 2xSCCT+50% formamide at 60°C for 20 minutes. Liquid was replaced with a hybridiziation mix: 50 μl formamide, 25 μl 4x hybridization buffer (40% dextran sulfate, 8xSSC, 0.8% Tween-20), 2 μl 20 μg/μl RNase A, 10 μM oligopaint probes labeled with Alexa 594, and ddH$_2$O added to reach a total volume of 100 μl. Denaturation at 90 ^{o}C for 3 min was followed by overnight hybridiziation at 37°C. Hybridization was followed by the following wash steps: four times with preheated 2xSSCT at 60°C and twice with 2xSSCT at room temperature, 5 min incubation time for each step. Before proceeding with the immunofluorescence protocol, the samples were additionally washed three times with 1 ml PBST for 5 minutes. Samples were blocked with 4% BSA in PBST, 30 minutes at room temperature, followed by incubation of primary antibodies (mouse anti-Pol II Ser5P (4H8, 1:300) and rabbit anti-Pol II Ser2P (EPR18855, 1:300)) in 4% BSA-PBST overnight at 4°C. Samples were washed three times for 5 min with PBST, once with 4% BSA-PBST, and again incubated overnight, 4°C with secondary antibodies (goat anti-mouse conjugated with STAR RED (1:300) and goat anti-rabbit conjugated with Alaxa 488 (1:300)) in 4% BSA-PBST. Finally, samples were washed three times with 1 ml PBST and mounted in Vectashield H-1000 under #1.5 selected cover glass.

An overview of the oligopaint probe sets is shown in table S3. Full oligo sequences and scripts used in probe design are provided as a supplementary file. The raw image data and analysis scripts are provided in the form of Zenodo repositories, see Data Availability statement.

Instant Structured Illumination Microscopy (instant-SIM)

Microscopy data from live human cheek cells, live and fixed zebrafish embryos were recorded using a commercial implementation of the instant-SIM high-speed super-resolution confocal microscopy principle (VisiTech iSIM) [33]. The microscope was built on a Nikon Ti2-E stand. For live imaging a Nikon Silicone Immersion Objective (NA 1.35, CFI SR HP Plan Apochromat Lambda S 100XC Sil) and for fixed imaging a Nikon Oil Immersion Objective (NA 1.49, CFI SR HP Apo TIRF 100XAC Oil) were used. Laser at 405 nm, 488 nm, 561 nm and 642 nm were used for excitation. The acquisition settings were kept constant across all samples of a given experimental repeat.

Optimizing the exposure time for high-speed time-lapse imaging: For the exposure times 150 ms, 100 ms, 70 ms, 40 ms or 20 ms, phase A images (short, long, long, short, short) were recorded, in which "long" corresponds to an exposure time of 200 ms, followed by a time-lapse with the different exposure times (phase B) for a total duration of 2 minutes (see table S1). For each exposure time, images from three embryos were recorded, from different nuclei for every time-lapse.

Condition	Exposure time	Full z-stack interval	Number of z-stacks
High-quality	200 ms	6 s	21
Shortened	100 ms	3 s	41
Shortened	50 ms	2 s	61
Shortened	20 ms	1 s	121
Shortened	10 ms	1 s	121

Table S1. Time-lapse parameters for high-speed imaging of Pol II clusters in live zebrafish embryos. Total duration of time-lapse was 2 min.

Noise2Void processing of STED microscopy data

For each nucleus, a pair of low-quality images a high-quality image were acquired. We first trained an n2v-network on the low-quality images and reconstructed both low-quality images using this n2v-network.

Noise2Void processing of cheek cell microscopy data

For each acquired position, we trained an n2v-network on the first low-quality image and reconstructed both low-quality images with the trained network. Procedures were reviewed and accepted by the Karlsruhe Institute of Technology ethics committee. Raw image data were stored in an anonymous fashion and are not for public release.

Metrics for image assessment

The SSIM metric for the comparison of images x and y is given by

$$SSIM(x,y) = \left\{ \frac{2\mu_x\mu_y + C_1}{\mu_x^2 + \mu_y^2 + C_1} \right\}^\alpha \times \left\{ \frac{2\sigma_x\sigma_y + C_2}{\sigma_x^2 + \sigma_y^2 + C_2} \right\}^\beta \times \left\{ \frac{2\sigma_{x,y} + C_3}{\sigma_x\sigma_y + C_3} \right\}^\gamma,$$

where C_1, C_2 and C_3 are constants with the default value of 0.01 and 0.03, $C_2/2$ respectively. With three constant α, β and γ (default values are $(1,1,1)$), the contribution of each term can be defined. μ_x and μ_y are the mean over each of the images' pixel intensities, σ_x and σ_y are the standard deviations, and $\sigma_{x,y}$ the covariance of the two images' pixel intensity values. The first, second and the third term are respectively called luminance (mean), contrast (standard deviation) and structural (covariance). As the standard deviation of an image is not usually affected by denoising [46], in our experiment, we only focused on luminance and structural term and we investigated how luminance and structural terms of SSIM depend on the duration of photon collection in integrating versus averaging mode detectors. We observed that, for integrating detectors, the mean term influences the SSIM value, so that structural reliability cannot be directly compared if this term is included in the calculation of the SSIM value (Fig. S1A-D). For SSIM and local SSIM analysis, we therefore only consider the structural term, given by

$$SSIM(x,y) = \frac{2\sigma_{x,y} + C_3}{\sigma_x\sigma_y + C_3},$$

In local SSIM experiments, we used a Gaussian kernel with standard deviation of 12 pixels for weighting the neighborhood pixels around a pixel.

The Fourier ring correlation (FRC) analysis is based on the cross-correlation of two images in frequency space, and relies on the assumption that the two images are two independent reconstructions of the same object with independent noise realizations. The spatial frequency spectra of the two images are first divided into bins, which are in turn sorted by location within ring-shaped regions relative to the center of the Fourier spectrum polar coordinates. The FRC curve then is calculated based on the cross-correlation of the power values over all bins for a given ring radius, r, as follows:

$$FRC(r) = \frac{\sum_{r_i \in r} F_1(r_i) \cdot F_2(r_i)^*}{\sqrt{\sum_{r_i \in r}(F_1(r_i))^2 \cdot \sum_{r_i \in r}(F_2(r_i))^2}}.$$

where F_1, F_2 are the Fourier transforms of two images and r_i refers to all frequency space bins that fall within a given ring radius r. The cut-off spatial frequency is the smallest r value for which the $FRC(r)$ value drops below the widely used threshold value of 1/7. Up to the spatial frequency given by $1/r$, the object is considered to be reliably resolved.

Analysis of morphology fluctuations in RNA polymerase II clusters

Input images are recruited RNA polymerase II (Pol II Ser5P) and elongating RNA polymerase II (Pol II Ser5P) in live zebrafish embryos visualized with antibody fragments (Fab) labelled with Janelia Fluor (Kimura Lab, Tokyo Tech), consisting of two channels, Pol II Ser2P and Pol II Ser5P, recorded by our proposed phase-AB imaging protocol (Fig. 3) with phase B spanning a time of 2 min. Data are recorded for four different exposure times ($t_{exp} = 10, 20, 50, 100$ ms) (Fig. S4A).

Noise2Void denoising We run the n2v-script on Google Colab[1] for the Pol II Ser5P channel for every exposure time for channel 2 (Pol II Ser5P) images. The patches which are given to the network are of size (16, 64, 64), 4/5 of patches were used to train the network and 1/5 of patches were used for validation. We trained the network on each image for 70 epochs with neighbourhood radius 10. After assessing the n2v-processed images with FRC and local SSIM (Fig. S4), time-lapse Ser5P series recorded by 20 and 50 ms exposure time were selected and reconstructed using the trained network. The Pol II Ser2P time-lapse series were left unchanged.

Median filter After n2v-reconstruction of Pol II Ser5P images, to recover the signal a median filter of size 50×50 pixel is applied to both Pol II Ser2P and Poll Ser5P images, and the filtered images are subtracted from the unfiltered images to remove local background fluctuations.

3D segmentation We used the MatLab adaptive threshold function with neighbourhood size $[101, 101]$ to segment each focus of Pol II Ser5P images in three dimensions. The adaptive threshold is calculated based on the local mean intensity (first-order statistics) in the neighbourhood of each pixel.

Foci tracking Each focus is tracked over time based on the minimal distances between foci in consecutive frames (Euclidean distance metric). Tracking was continued if a focus disappeared at t_n and again appeared at t_{n+1}, this bridging single frame disappearance events. We extracted foci that could b tracked for longer than 70 s for further analysis.

[1]https://colab.research.google.com/drive/1KLJcvng36JiPlGH2GWaO2VxR2bppJ0xc?usp=sharing

2D segmentation We then picked the middle z-plane of each focus at each time point and segmented Pol II Ser5P in this plane by the adaptive threshold algorithm (Fig. 4B and Fig. S6A). The algorithm computed a locally adaptive threshold for each pixel based on the neighborhood pixels of size (25 * 25).

Shape quantification For each focus, we reported four properties (Ser2P mean intensity, Ser5P mean intensity, solidity, area and elongation) (Fig. 4C and Fig. S6B). If the focus lost at t_n and again appeared at t_{n+1}, the average quantification of the focis at t_{n-1} and t_{n+1} is instead considered. The elongation is calculated by the division of the major axis and the minor axis of the focus. For the calculation of Ser2P mean intensity, the corresponding Ser5P segmented area in Ser2P images is considered. For Ser5P intensity property, the intensities of central pixels (of size 9×9 pixels) is averaged.

Correlation analysis Time-shifted ($[-20\,\mathrm{s}, 20\,\mathrm{s}]$) cross correlation between Ser5P intensity and elongation, Ser2P intensity and elongation, and solidity and elongation are quantified (Fig. 4D and Fig. S6C).

Pseudo-time analysis from single time point fluorescence images

In this analysis, we work with still images of Pol II clusters, which were obtained from fixed zebrafish embryos. While these do not allow tracking Pol II clusters over time, the additional oligopaint fluorescence label can be used as information to sort images along a hypothetical timeline. The pseudo-time analysis used for this sorting approach works under the assumption that, in cases where genes engage with Pol II clusters for transcriptional activation, a stereotyped sequence of events occurs that includes changes in Pol II phosphorylation, cluster shape changes, and association of the gene with a given cluster. Based on this assumption, the goal of this analysis is to reproduce such a stereotypical sequence by sorting Pol II cluster-gene pairs detected from an ensemble of still images

Nuclei were segmented by Otsu thresholding of blurred (Gaussian blurring, $\sigma = 1.0\,\mu\mathrm{m}$) and background-subtracted (after Gaussian blurring, $\sigma = 10\,\mu\mathrm{m}$) images. Only nuclei with a volume greater than $40\,\mu\mathrm{m}^3$ and a solidity greater than 0.7 were retained for further analysis. Pol II clusters were segmented inside each nucleus separately by robust background thresholding (2 standard deviations above intensity mean) after background subtraction ($3.0\,\mu\mathrm{m}$) from the Pol II Ser5P channel. Only Pol II clusters with a volume greater than $0.03\,\mu\mathrm{m}^3$ were retained for further analysis. Oligopaint-labeled genes were detected by robust background thresholding (6 standard deviations above intensity mean) of smoothed (Gaussian blurring, $\sigma = 100\,\mathrm{nm}$) and background-subtracted (Gaussian blurring, $\sigma = 5\,\mu\mathrm{m}$) images of the oligpaint channel. Only objects with a volume greater than $0.05\,\mu\mathrm{m}^3$ were retained for further analysis.

Gene-cluster interactions should be detectable by spatial proximity of a given gene to a Pol II cluster. The analysis thus connects any detected gene to the nearest neighboring Pol II cluster (Euclidean distance, Fig. S8B), resulting in a cluster-gene pair. These cluster-gene pairs are from here on treated as single observations, the remaining task is to sort these cluster-gene pairs into a coherent sequence. Only gene-cluster pairs with a cluster volume greater than $0.2\,\mu\mathrm{m}^3$ were retained for further analysis.

The sorting of cluster-gene pairs is based on a mapping of correlated changes in the properties of cluster-gene pairs. Specifically, each cluster-gene pair is represented as a point an 8-dimensional feature space ($\mathcal{R}^8$) defined by the gene and cluster properties (S8B). Application of a principal component analysis (PCA) to this ensemble of $\mathcal{R}^8$ coordinates allows an effective reduction of dimensionality, and provides a mapping into distinct regions in the space spanned by the two first principal components (Fig.

S8C). Plotting the projections of cluster volume and gene Ser5P level into this PCA plot, an orthogonal coordinate system can be defined (Fig. S8C). Using only linear transformations (rotation and reflection), the cluster volume can be used as one axis of the coordinate system, and the gene Pol Ser5P placed to the left side of the plot. Inside this plot, data points can now be directly sorted in clock-wise order, providing a pseudo-time sorted dataset (Fig. S8C). This pseudo-time sorting is only successful when a gene engages in close contact with Pol II clusters with an increased frequency (centroid-centroid distance threshold for contact detection of 200 nm); genes that only engage Pol II clusters less often do not exhibit a useful pseudo-time sorting (Fig. S8D).

For the pseudo-time-sorted data points, a periodic progress coordinate $s \in [0, 1)$ can be defined, assigning to each data point i a coordinate

$$s = n_i/N,$$

where n_i is the pseudo-time sorted index of the data point i in a data set consisting of a total of N data points. This progress coordinate can, in turn, be used to carry out cross-correlation analyses, based on a pseud-time shift Δs (Fig. S9). For genes with high frequencies of engagement with Pol II clusters, this cross-correlation analysis closely reproduces the relationship between cluster morphology and Pol II Ser5 phosphorylation seen in our live imaging experiments (Fig. S9 *foxd5*, *klf2b*, *zgc::64002*). For genes with lower frequencies of engagement, the clear patterns of correlation between cluster elongation and Pol II Ser5 phosphorylation are not detected (Fig. S9 *vamp2*, *ripply1*, *drll.2*, *gadd45ga*, *iscub*).

Oligopaint library design

Staining of a repetitive region to establish fluorescence in-situ hybridization

To test the FISH procedure without the need of a full oligopaint library, a single probe against a repetitive region was designed. The zebrafish genome was screened for long repetitive regions using BLAST. A region containing 100 repeats on chromosome 25 was selected (repeat sequence: 5'- CCGACGCATCTTCGTGCTGG CTTACATACTC-CGCTGCACC AATGACTTGAATTGCAGCCT TGGGCGTATGCTGCTC). Probes were produced by the same protocol used for actual oligopaint production, using a primer Alexa Fluor 594-conjugation at the 5' end (Thermofisher). Primer sequences are shown in Tab. S2).

Primer	Sequence 5' to 3'	Melting temp.
Chr25-Forward	CCGACGCATCTTCGTGCTGG	53°C
Chr25-Reverse with T7 promotor sequence	TAATACGACTCACTATAGGGG AGCAGCATACGCCCAAGGC	65°C
Chr25-Forward with Alexa 594	Alexa594-CCGACGCATCTTCGTGCTGG	70°C

Table S2. Primers used for PCR. Sequence (5' to 3') and melting temperature of each primer.

Oligopaint probe library

Genes were chosen based on their Pol II Ser5P ChIP-Seq signal from Zhang et al. 2014 [75]. For each gene, Pol II Ser5P ChIP-Seq peaks in the promoter region (2 kb region upstream from the gene) were called using MACS2 [76]. For each peak, a p-value, q-value (Benjamini-Hochberg corrected p-value) and a signal value (fold enrichment of peak against background) were calculated. The peaks with p-value $< 10^{-5}$, q-value $< 10^{-4}$ and signal value > 3 were chosen. For these genes RNA-Seq data from White et al. 2017 were compared [77]. Only genes without high maternally provided RNA levels were considered, as indicated by low RNA counts for developmental stages preceding the high stage. Further, genes were hand-picked to cover a range of different RNA counts at the sphere stage. The scripts used for the gene selection process are provided as a supplementary file. Oligopaint libraries were designed using the OligoLego program [78]. Sequences of 32 nucleotides were mined from the zebrafish genome and are provided by the program (Tab. S3). Each probe set was designed to cover approximately 25 kb upstream and 25 kb downstream the gene, with density of 4 probes/kb where possible. The oligopaint probe library was ordered from Twist Bioscience. The text file used to order the oligo pool is attached as a supplementary file. Associated amplification primers are listed in Tab. S4.

Data availability

Scripts and raw data are available at the following URLs.

Microscopy data and scripts for analysis of local SSIM and FRC in live zebrafish embryos: https://doi.org/10.5281/zenodo.5568871

Microscopy data and scripts for FRC comparison between low-quality images, reconstructed images, deconvolved images, and high-quality images: https://doi.org/10.5281/zenodo.6382528

Python scripts for assessment of different SSIM terms for integrating vs. averaging photon collection: https://doi.org/10.5281/zenodo.5569195

Scripts for SSIM and Fourier ring correlation analysis of Noise2Void-reconstructed STED images: https://doi.org/10.5281/zenodo.5569432

Microscopy data of RNA Pol II CTD phosphorylation in live zebrafish embryos: https://doi.org/10.5281/zenodo.5566880

Scripts used for morphology fluctuations analysis in Pol II clusters: https://doi.org/10.5281/zenodo.5569475

Matlab scripts used in the analysis of oligopaint-immunofluorescence image data: https://doi.org/10.5281/zenodo.5524939

Microscopy data for the gene *drll.2*: https://doi.org/10.5281/zenodo.5266592

Microscopy data for the gene *iscub*: https://doi.org/10.5281/zenodo.5266736

Microscopy data for the gene *vamp2*: https://doi.org/10.5281/zenodo.5266903

Microscopy data for the gene *gadd45ga*: ttps://doi.org/10.5281/zenodo.5268538

Microscopy data for the gene *foxd5*: https://doi.org/10.5281/zenodo.5266995

Microscopy data for the gene *klf2b*: https://doi.org/10.5281/zenodo.5268833

Microscopy data for the gene *ripply1*: https://doi.org/10.5281/zenodo.5268779

Microscopy data for the gene *zgc::64022*: https://doi.org/10.5281/zenodo.5268683

Genes	Labeled length	Number of probes
foxd5	158 kb	804
klf2b	250 kb	816
zgc:64022	252 kb	810
vamp2	160 kb	845
ripply	190 kb	808
drll.2	210 kb	862
gadd45ga	180 kb	845
iscub	120 kb	837

Table S3. Genes chosen for oligopaint DNA FISH, with the size of the labeled genomic region and number of oligopaint probes covering the gene region.

Primer	Sequence<br>5' to 3'	Melting<br>temp.
Universal Forward	CGGCTGCCGCTAAGAGTCTC	53°C
Forward with<br>Alexa Fluor 584	Alexa594-CCGACGCATCTTCGTGCTGG	63.5^{o}C
Universal Reverse	GAGCAGCATACGCCCAAGGC	53°C
foxd5	GGGAGGGTGTGTGGTCGCTT	53°C
klf2b	CCGAGGCGCATGTGTATCCC	53°C
zgc:64022	CTGATTCGACCCGCCCTGGA	53°C
vamp2	GTCTGCCACCCTTCGCATGG	53°C
ripply1	GGCTCCAGCTAACACGCGGA	53°C
drll.2	CTCCGTACTGCCGCGATTCC	53°C
gadd45ga	CGCATTCATCCACCGCCGAT	51°C
iscub	GGGTCGCTACAAGTGCGCTC	53°C

Table S4. Sequence (5' to 3') of primers used for PCR for chosen genes for oligopaint FISH and according melting temperatures.

List of Supplementary Figures:

Fig. S1, page 12
Fig. S2, page 13
Fig. S3, page 14
Fig. S4, page 15
Fig. S5, page 16
Fig. S6, page 16
Fig. S7, page 17
Fig. S8, page 18
Fig. S9, page 19

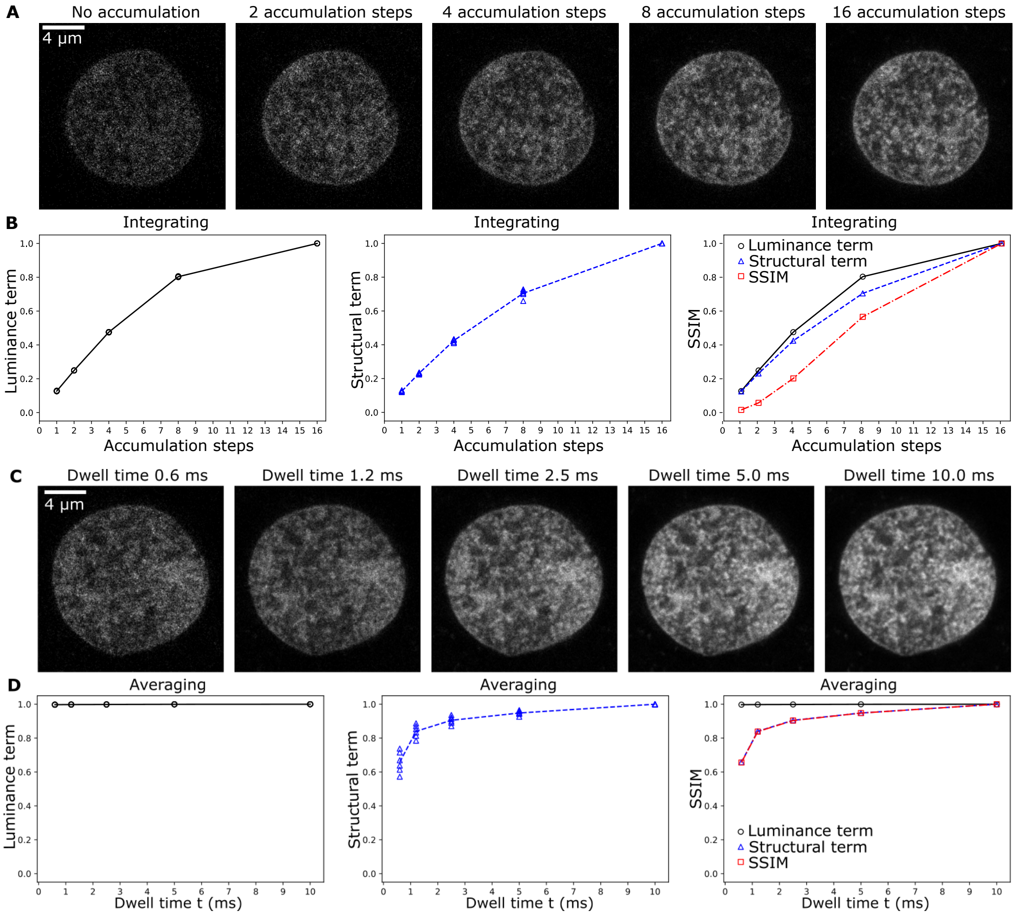

Fig S1. Structural similarity is a metric of proper structure. Figures show the DNA distribution in a mid-section of a nucleus in a fixed zebrafish embryo, obtained by stimulated emission depletion (STED) microscopy. A) Depending on the microscope detector type and settings, photons can be accumulated over time without normalization (integrating detector) or with normalization (averaging detector). To illustrate how the different terms of the structural similarity index metric (SSIM) depend on the duration of photon collection in the integrating type detector, images were acquired with line-repeat scans in the accumulation mode, using increasing numbers of line repeats $(1, 2, 4, 8, 16)$. B) In the integrating detector case, the luminance (mean) as well as the structural (covariance) term of the SSIM depend on the number of accumulation steps. Overall SSIM values contain contributions from both terms, so that an assessment of structural reliability would be obscured by changes in overall image intensity. SSIM values were calculated based on $n = 6$ images obtained by a reduced number of accumulation steps and a reference image obtained with the highest number of accumulation steps (16). Individual values are shown with the mean. C) To illustrate how the different terms of the SSIM metric depend on the duration of photon collection in the averaging detector type, DNA images were acquired with the detector left open to collect photons at each pixel for longer times (dwell time t), then the photon count is normalized by the dwell time t. D) In the integrating detector case, the luminance term is constant and close to the value of 1.0. Only the structural term changes with increasing t, so that also the overall SSIM values directly reflect structural reliability for a given t. SSIM values were calculated based on $n = 6$ images obtained for a given t, combined with a matching image recorded with the highest $t = 10\,\text{ms}$. Individual values are shown with the mean.

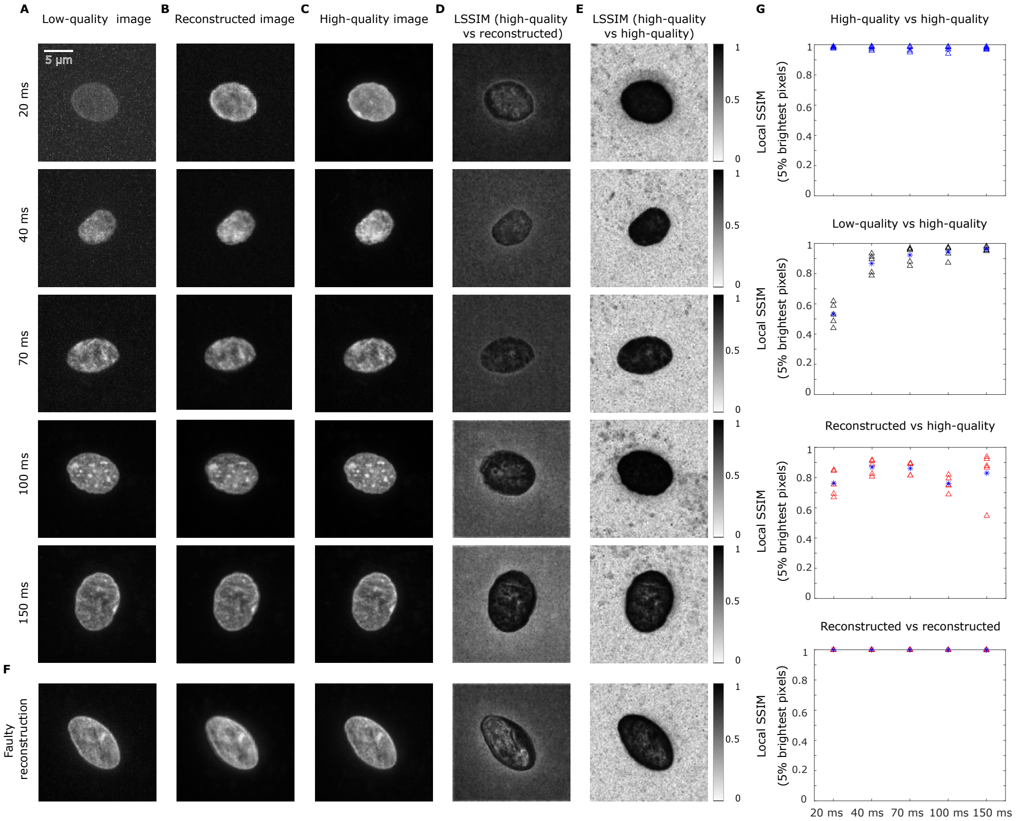

Fig S2. Local structural similarity index metric can show faulty reconstructions. A) Representative micrographs of nuclei of human cheek cells in which DNA was labelled by Hoechst 33342. Images are maximum intensity projections of full volumetric stacks acquired with different exposure time (t_{exp}) as indicated B) Noise2Void-processed images corresponding to panel A. C) High-quality images acquired at the same position, but with $t_{exp} = 200$ ms. D) Local structural similarity index metric (SSIM) map for the comparison between reconstructed images and high-quality images. E) Local SSIM map for the comparison between two high-quality images acquired at the same position, suggesting that there is no structural mismatch in the area of interest. F) An example of a faulty reconstruction, indicated by a structural mismatch inside the area of interest. G) Average SSIM values based on 5% lowest local SSIM value of the 5% brightest pixels. $n = 4, 5, 5$ values from $N = 5$ different nuclei.

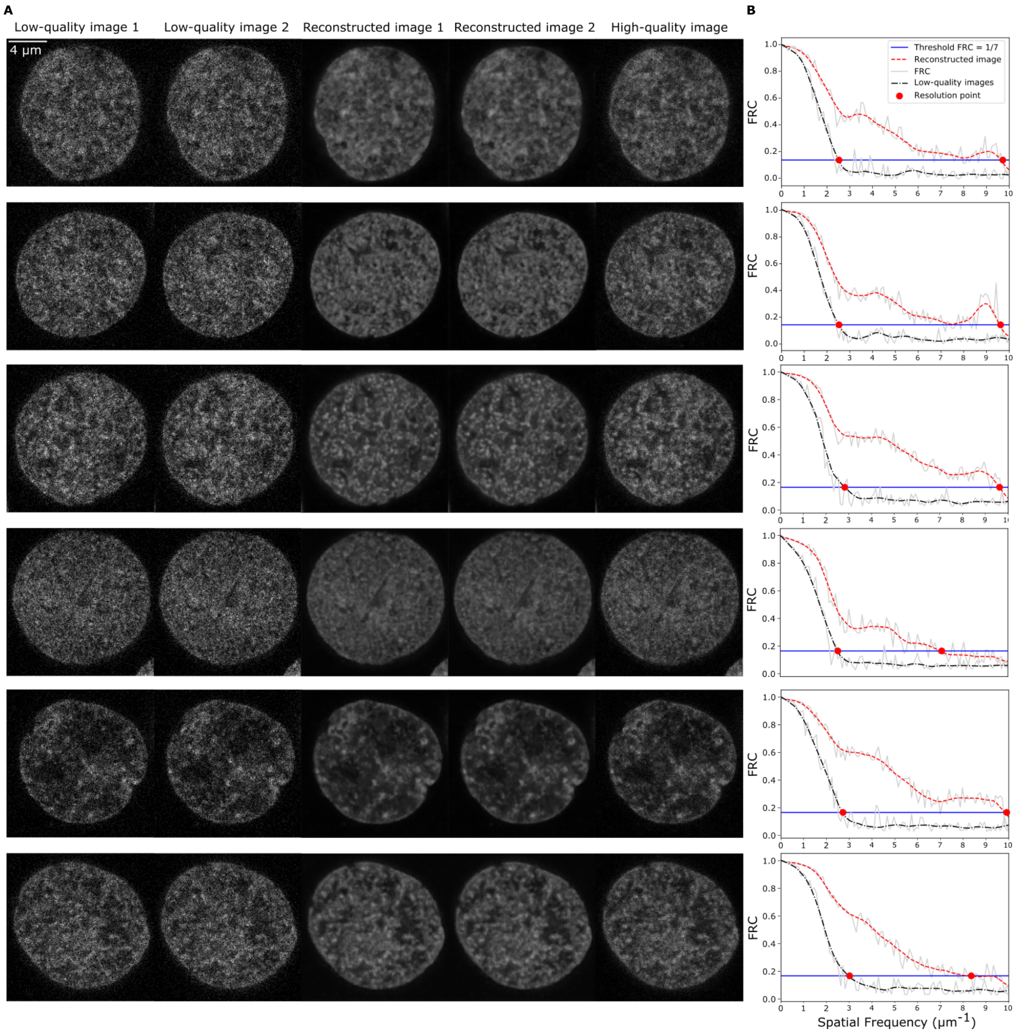

Fig S3. Fourier ring correlation can quantify improvements in effective image resolution obtained by Noise2Void reconstruction. A) DNA distribution in mid-sections of nuclei in a fixed zebrafish embryo, obtained by stimulated emission depletion (STED) microscopy. Low-quality image 1 and 2 are acquired with identical acquisition settings. Reconstructed image 1 and 2 are obtained by Noise2Void reconstruction from the low-quality images 1 and 2, respectively. The high-quality image was acquired in the same scanning sequence as the low-quality images, but included accumulation by repeated line-scanning to improve image quality. B) Fourier ring correlation (FRC) analysis to determine the improvement in effective resolution of reconstructed images relative to the unprocessed low-quality images.

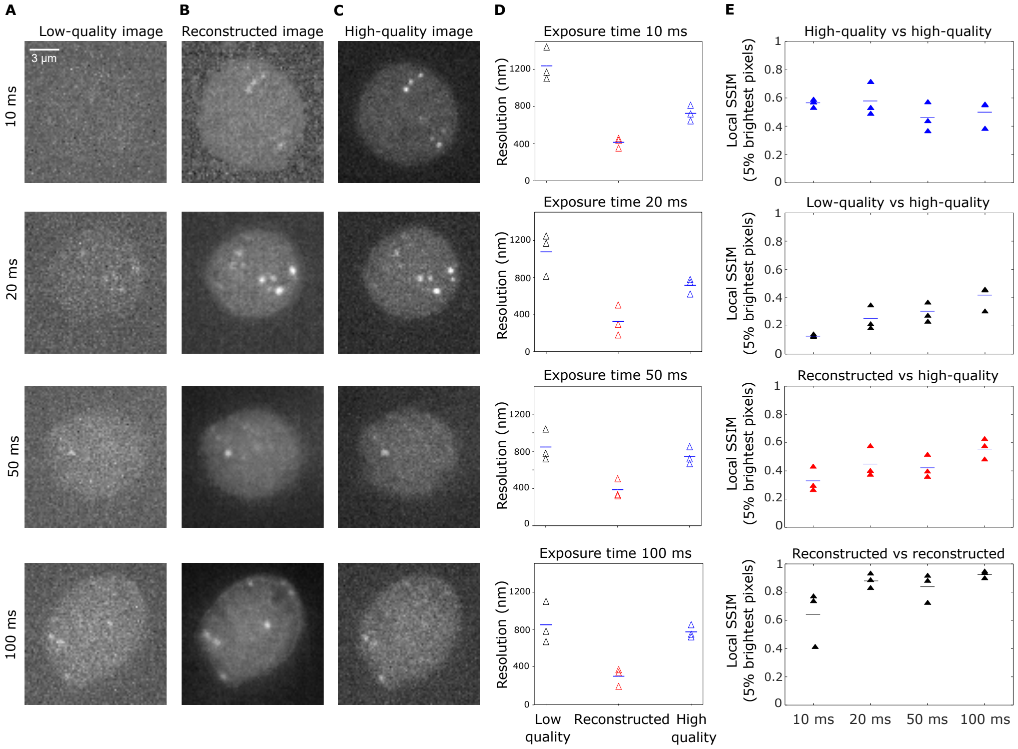

Fig S4. **Metric-based assessment to show how far the signal-to-noise of the image can be compromised while still allowing Noise2Void to recover signal-to-noise ratio post-acquisition.** A) Representative micrographs of recruited RNA polymerase II (Pol II Ser5P) in live sphere-stage zebrafish embryos, visualized with antibody fragments (Fab) labelled with Janelia fluor 647. Images are single image planes and were acquired with different exposure times t_{exp} as indicated. Intensity scale from black to white adjusted to the 0.01-th and the 99.99-th percentile B) Corresponding images after Noise2Void-based reconstruction. C) Corresponding high-quality reference images captured with an t_{exp} of 200 ms. D) Effective resolution as determined by FRC analysis for low-quality images, reconstructed images, and high-quality images for the indicated t_{exp}. $n = 4, 3, 3$ values from $N = 3$ different embryos are shown with the mean. E) Average SSIM values based on the 5% lowest local SSIM values of the 5% brightest pixels. $n = 4, 4, 3$ values from $N = 3$ different embryos are shown with the mean.

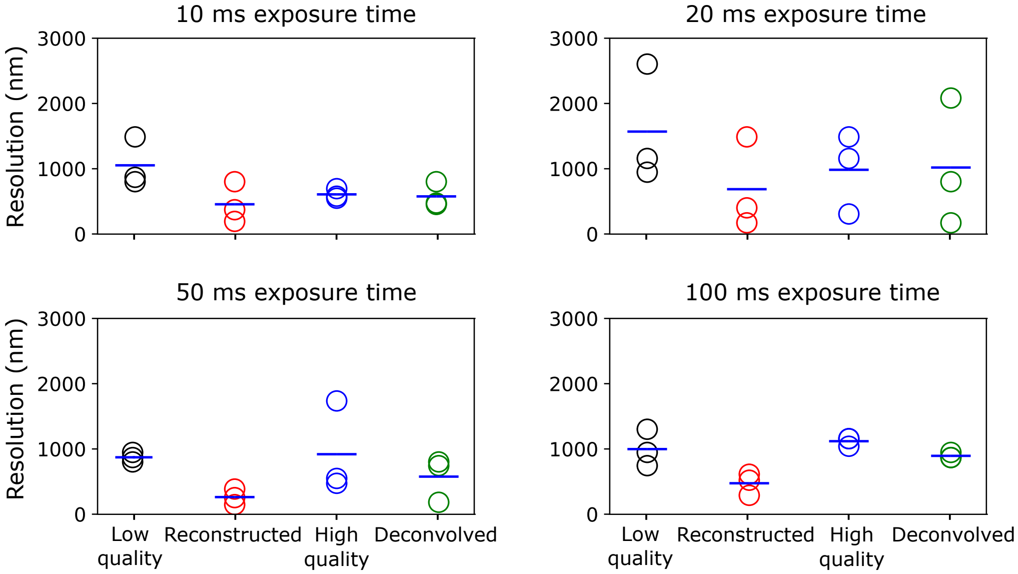

Fig S5. Comparison of improvement of effective resolution by Noise2Void and conventional 3D deconvolution. Effective resolution as determined by FRC analysis for low-quality images, reconstructed images, high-quality images and 3D deconvolved images for the indicated t_{exp}. $n = 4, 4, 3$ values from $N = 3$ different embryos are shown with the mean. Deconvolved images were obtained by Richardson-Lucy 3D deconvolution.

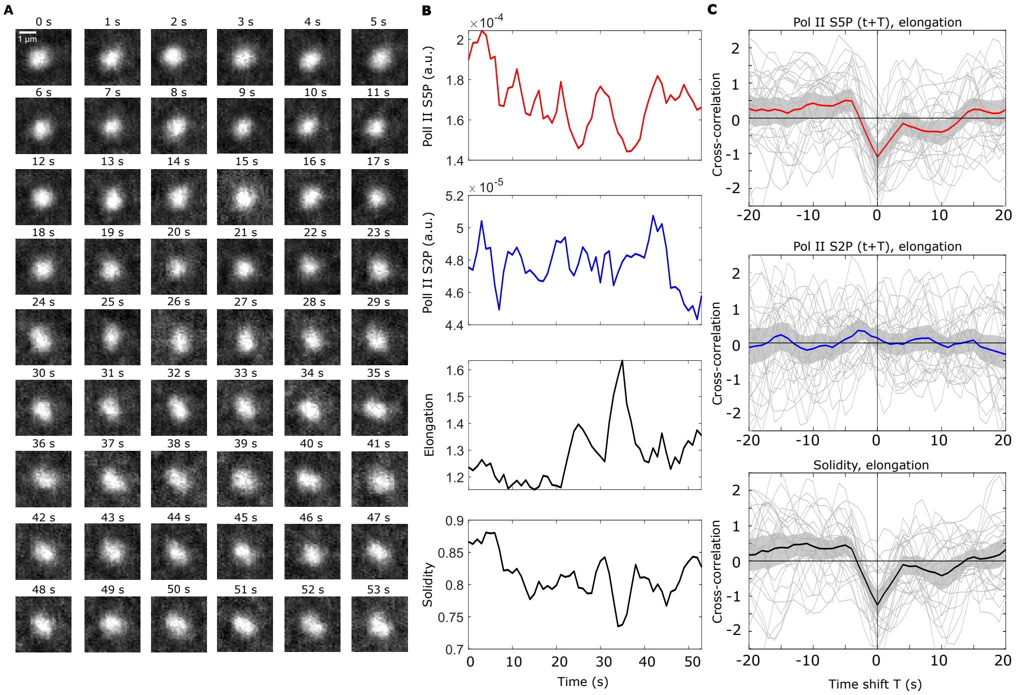

Fig S6. Coordinated changes in RNA polymerase cluster phosphorylation and shape are reproduced by Noise2Void-accelerated imaging with a different exposure time. A) Representative series of time-lapse images showing a single RNA polymerase II cluster in the Pol II Ser5P channel (single image plane from the middle z position of the cluster, exposure time $t_{exp} = 20\,\text{ms}$, effective time resolution for acquisition of full 3D volumes 1 s). B) Time courses of the Pol II Ser5P intensity, the Pol II Ser2P intensity, elongation, and solidity for the example track shown in panel A. C) Cross-correlation analysis, gray lines indicate the analysis results for individual cluster time courses, thick lines the mean over all analyzed clusters, the gray region is the bootstrap 95% confidence interval. Analysis based on $n = 27$ clusters, recorded from one sphere-stage embryo.

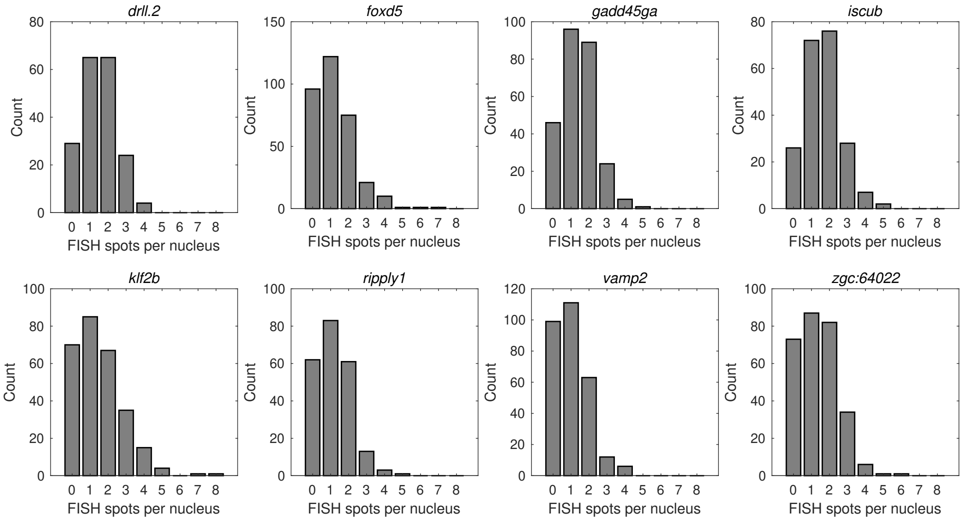

Fig S7. **Number of foci detected by oligopaint fluorescence in-situ hybridization.** Number of foci detected per cell nucleus in the channel representing oligopaint fluorescence in-situ hybridization (FISH) signal for the genes indicated above each graph.

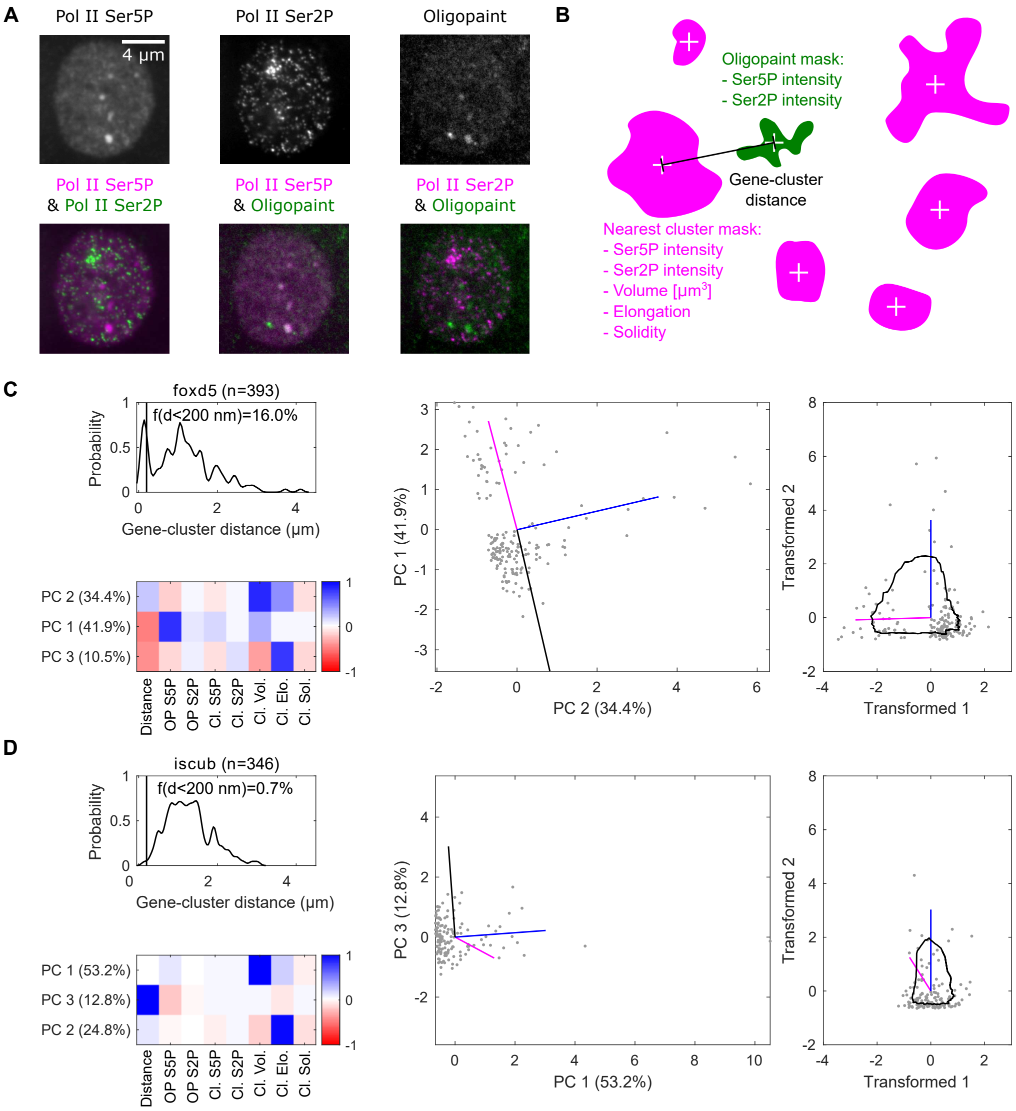

Fig S8. **Single images of clusters from fixed embryos can be sorted in pseudo-time based on their interaction with visiting genes.** A) Example micrographs of a nucleus of a fixed sphere-stage zebrafish embryo with Pol II Ser5P, Pol II Ser2P, and oligopaint fluorescence *in situ* hybridization (target gene *foxd5*) signal. B) Sketch of properties extracted from Pol II Ser5P cluster-oligopaint nearest neighbor pairs. C) Overview of the pseudo-time reconstruction procedure in the case of a gene with a high frequency of visiting Pol II Ser5P clusters (quantified as the fraction f of observations with less than 200 nm distance between the oligopaint signal and the nearest Pol II Ser5P cluster, d). The top three principal component (PC) support vectors are displayed (top two PC vectors are sorted so that the top vector has the higher weight in the volume dimension). The two top vectors are used to plot single observations in a two-dimensional overview plot. A rotation and inversion transformation are then applied to ensure that always the volume points exactly North, and the gene Pol II Ser5P intensity towards the West half of the graph. Values can now be sorted according to angle relative to the direction North, and a running average over these angles indicates how well the sorted values are distributed away from the coordinate origin. In the case of the gene *foxd5*, a clear separation away from the origin can be seen, indicating successful sorting by pseudo-time. D) In the case of the gene *iscub*, which does not frequently associate closely with Pol II Ser5P clusters, the individual points form a single cloud close to the origin and the running average line is also close to the coordinate origin, indicating that the attempt of sorting by a pseudo-time coordinate is not successful.

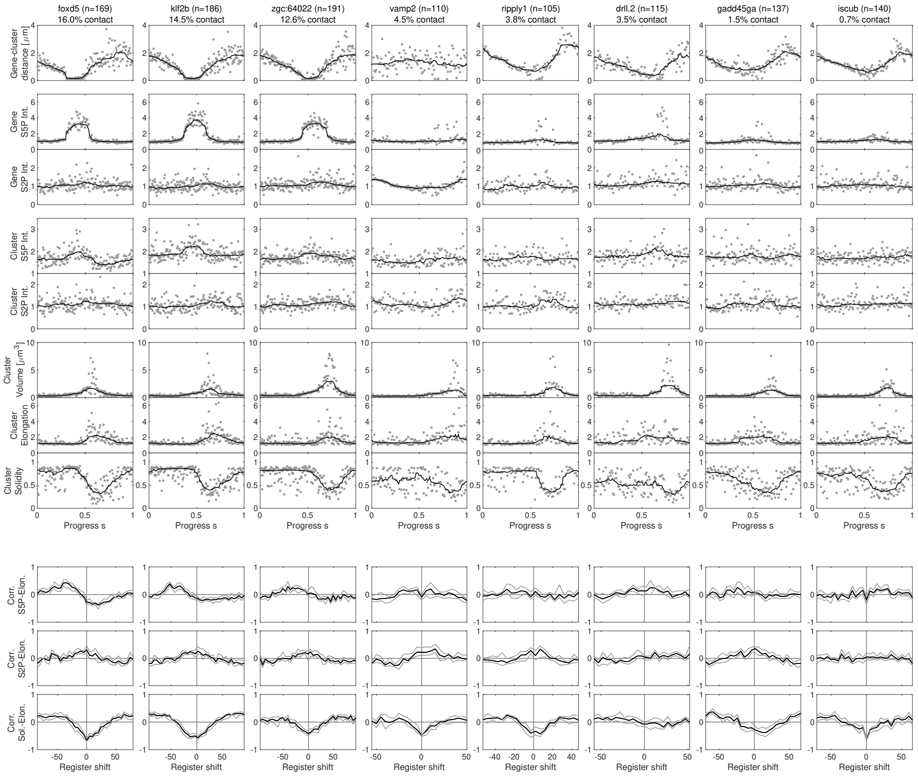

Fig S9. **Pseudo-time sorting reproduces correlation analysis results only for genes that frequently associate with RNA polymerase II clusters.** Results of the pseudo-time sorting for eight genes that were labeled oligopaint fluorescence *in-situ* hybridization. Percentage of contact (f) is calculated as the percentage of OP-cluster pairs with distance d of 200 nm or less. Shown are the pseudo-time sorted oligopaint-cluster distance, the Pol II Ser5P and Ser2P signals at the oligopaint-labeled gene (Gene S5P Int., Gene S2P Int., normalized against the whole nucleus median intensity), the Pol II Ser5P and Ser2P intensity at the nearest Pol II Ser5P cluster (Cluster S5P Int., Cluster S2P Int.), cluster volume, cluster elongation, and cluster solidity. The coordinate s represents the progress in pseudo-time. A register shift in pseudo-time was used to calculate the cross-correlation between cluster Pol II Ser5P intensity and elongation, cluster Pol II Ser2P intensity and elongation, and cluster solidity and elongation. Number of cluster-gene pairs included in the analysis indicated as n for each gene. For each gene, images were recorded from four samples, distributed over two independent experiments.
